# Circulating acyl and des-acyl ghrelin levels in obese adults: a systematic review and meta-analysis

**DOI:** 10.1038/s41598-022-06636-3

**Published:** 2022-02-17

**Authors:** Yanmei Wang, Qianxian Wu, Qian Zhou, Yuyu Chen, Xingxing Lei, Yiding Chen, Qiu Chen

**Affiliations:** 1 Hospital of Chengdu University of Traditional Chinese Medicine, No. 39 Shi-er-qiao Road, Jinniu District, Chengdu, 610075 Sichuan China; 2 Ya‘an Polytechnic College, No. 130 Yucai Road, Yucheng District, Yaan, 625000 Sichuan China; 3 Halifa Regional Centre for Education, No. 33 Spectacle Lake Dr, Dartmouth, NS B3B1X7 Canada

**Keywords:** Biochemistry, Biomarkers, Endocrinology, Health care

## Abstract

Ghrelin is the only known orexigenic gut hormone, and its synthesis, secretion and degradation are affected by different metabolic statuses. This meta-analysis aimed to investigate the potential differences in plasma acyl ghrelin (AG) and des-acyl ghrelin (DAG) concentrations between normal weight and obese adults. Systematic literature searches of PubMed, Embase and Web of Science through October 2021 were conducted for articles reporting AG or DAG levels in obesity and normal weight, and 34 studies with 1863 participants who met the eligibility criteria were identified. Standardized mean differences (SMDs) with 95% confidence intervals (CIs) were calculated to evaluate group differences in circulating AG and DAG levels. Pooled effect size showed significantly lower levels of baseline AG (SMD: − 0.85; 95% CI: − 1.13 to − 0.57; *P*_SMD_ < 0.001) and DAG (SMD: − 1.06; 95% CI: − 1.43 to − 0.69; *P*_SMD_ < 0.001) in obese groups compared with healthy controls, and similar results were observed when subgroup analyses were stratified by the assay technique or storage procedure. Postprandial AG levels in obese subjects were significantly lower than those in controls when stratified by different time points (SMD _30 min_: − 0.85, 95% CI: − 1.18 to − 0.53, *P*_SMD_ < 0.001; SMD _60 min_: − 1.00, 95% CI: − 1.37 to − 0.63, *P*_SMD_ < 0.001; SMD _120 min_: − 1.21, 95% CI: − 1.59 to − 0.83, *P*_SMD_ < 0.001). In healthy subjects, a postprandial decline in AG was observed at 120 min (SMD: − 0.42; 95% CI: − 0.77 to − 0.06; *P*_SMD_ = 0.021) but not in obese subjects (SMD: − 0.28; 95% CI: − 0.60 to 0.03; *P*_SMD_ = 0.074). The mean change in AG concentration was similar in both the obese and lean health groups at each time point (ΔSMD_30min_: 0.31, 95% CI: − 0.35 to 0.97, *P*_SMD_ = 0.359; ΔSMD_60min_: 0.17, 95% CI: − 0.12 to 0.46, *P*_SMD_ = 0.246; ΔSMD_120min_: 0.21, 95% CI: − 0.13 to 0.54, *P*_SMD_ = 0.224). This meta-analysis strengthens the clinical evidence supporting the following: lower baseline levels of circulating AG and DAG in obese individuals; declines in postprandial circulating AG levels, both for the healthy and obese individuals; a shorter duration of AG suppression in obese subjects after meal intake. These conclusions have significance for follow-up studies to elucidate the role of various ghrelin forms in energy homeostasis.

## Introduction

Obesity is defined by the Centers for Disease Control and Prevention (CDC)^1^ as a body mass index ≥ 30 kg/m^2^, and has become a global epidemic with the improvement of living standards. As the major risk factor for a large number of serious complications, individuals with obesity are more likely to have diabetes mellitus, dyslipidemia, hypertension, nonalcoholic fatty liver, cardiovascular disease, cancer and severe coronavirus disease 2019 (COVID-19), which lead to a higher rate of adult mortality^2,3^. Obesity is a multifactorial disease that is particularly associated with malfunctioning signal mechanisms. Complex signaling systems regulate energy homeostasis, where gastrointestinal hormones have a central physiological function.

Ghrelin is a gut hormone with the strongest orexigenic signal^4^ that helps the body respond to changes in metabolic status by binding to growth hormone secretagogue receptors (GHSRs) expressed in multiple central and peripheral targets^5,6^, with actions that include an increase in caloric intake, downregulation of energy expenditure^7^, potentiation of growth hormone (GH) release^8^, stimulation of gastric emptying and motility^6,9^, and anti-depressant-like properties^6^.

Endogenous ghrelin in adults is produced predominantly by P/D1 cells, which are located in the oxyntic glands of gastric funds^10,11^. Following secretion into the bloodstream, ghrelin circulates in two major forms: acyl ghrelin (AG) and des-acyl ghrelin (DAG, also known as unacyl ghrelin), and the ratio of the former to the latter is approximately 10% in plasma^12,13^. AG is a 28 amino acid peptide hormone often seen as the active form of ghrelin because of its unique posttranslational acylation at the serine 3 residue, which is catalyzed by ghrelin-O-acyltransferase (GOAT) and is essential for binding a GHSR with high affinity^14,15^. Without acylation, ghrelin can be secreted directly in the form of DAG. Furthermore, DAG is considered to be a degradation product of acyl ghrelin in the circulation due to esterase-catalyzed deacylation by multiple plasma proteins, especially acyl-protein thioesterase 1 (APT1)^16^ and butyrylcholinesterase (BChE)^17,18^. Although des-acyl ghrelin does not activate GHSR at physiological ranges^11^, emerging evidence has shown its independent biological activity, which may antagonize the orexigenic effects of acyl ghrelin in some instances^19–22^ even if the receptors and mechanisms remain undefined^20,23–26^.

The synthesis, secretion and degradation of ghrelin are affected by different metabolic statuses^27^. Current knowledge regarding circulating ghrelin levels describes that circulating ghrelin levels elevated during short-term fasting and decreased upon meal ingestion in healthy humans^6,28,29^, which is consistent with its unique mechanism of action on orexigenic hormone evolution for energy storage and seeking^30^. Thus, ghrelin is believed to increase the risk of obesity. Contrary to expectations, people with obesity usually exhibit lower fasting levels of ghrelin^31,32^ with a decline in postprandial suppression^33^, and different published studies that focus on obesity have reported a negative correlation between plasma ghrelin levels and body mass index^31,32^. This abnormality may result from physiological adaptation with a positive energy balance in obese participants^31^; however, it is important to note that merely evaluating total ghrelin could not reflect the real metabolic status in obesity, since acyl and des-acyl ghrelin interact with different receptors and appear to have opposite actions. The decrease in ghrelin may be attributed to the balance change between the two forms or significant DAG reduction. Given the rapid deacylation of AG to DAG by plasma esterases and the limitations of assay methods, the accurate assessment of circulating ghrelin has proven to be challenging, and only a few studies have assessed both forms independently. Following the commercialization of sandwich ELISA kits and the standardization of collection, handling and storage of biological samples, it is possible to distinguish and measure the two different forms correctly^34,35^. However, recent observations have shown inconsistent results in the circulating levels of AG and DAG in individuals with obesity, reflected not only at baseline but also at postprandial levels^36–44^.

Soon after its discovery, “the hunger hormone”^45^ attracted increasing interest in the treatment of obesity and related diseases. To date, GHSR antagonists, ghrelin vaccines and GOAT inhibitors have shown some promise for weight loss, calorie reduction and energy expenditure. Nonetheless, the maintenance of energy homeostasis through the ghrelin system is far more complicated than previously appreciated, and simply suppressing or reducing ghrelin has not achieved the desired treatment goal in human trials^46–49^.

Thus, a full understanding of the difference in the biochemical composition of plasma ghrelin under different dietetic states between normal weight and obese individuals is indispensable before the identification of pharmacological targets in ghrelin signaling. Therefore, the aim of this study was to conduct a meta-analysis of all eligible published articles to independently investigate the potential differences in plasma AG and DAG concentrations between normal weight and obese adults.

## Methods

The report and conduct of this systematic review and meta-analysis was based on the PRISMA (Preferred Reporting Items for Systematic Reviews and Meta-Analysis) statement, and a protocol has been registered in PROSPERO (International Prospective Register of Systematic Reviews) with the registration number CRD42021247253.

### Literature search

Literature searches were conducted based on three online electronic bibliographic databases, PubMed, EMBASE and Web of Science, from their date of inception up to 22 October 2021. We used Medical Subject Headings (MeSH) words of “obesity” and the free terms to represent the disease, the key words “acylghrelin” OR “acyl ghrelin” OR “acyl-ghrelin” OR “active ghrelin” OR “active-ghrelin” OR “acylated ghrelin” OR “acylated-ghrelin” OR “desacylghrelin” OR “desacyl ghrelin” OR “desacyl-ghrelin” OR “des-acyl ghrelin” OR “des-n-octanoyl ghrelin” OR “unacyl ghrelin” OR “unacyl-ghrelin” OR “unacylated ghrelin” OR “unacylated-ghrelin” OR “des-acylated ghrelin” OR “desacylated-ghrelin” OR “desacylated ghrelin” OR “non-aclyated ghrelin” OR “nonacylated ghrelin” OR “nonacylated-ghrelin” as our target. To ensure maximum eligible study coverage, the reference lists of pertinent articles were inspected manually. The full search strategies for all databases can be found in Supplementary Table 1. Two authors (YM Wang and QX Wu) independently screened and cross-checked the literature by identifying all titles and abstracts. Then, the selected articles were reviewed in full to ensure compliance with the inclusion criteria. A third author (Q Chen) was consulted regarding the disagreements. Specific libraries were created to allow the identification and exclusion of duplicate studies and the division and organization of the results.

### Selection criteria

(1) Articles studying the circulating acyl or des-acyl ghrelin levels in obese humans aged 18 to 80 years; (2) BMI was used to define obesity with the following standards^1^: normal weight: 18.5 to < 25 kg/m^2^, overweight: 25.0 to < 30 kg/m^2^, obesity: ≥ 30 kg/m^2^. Both overweight and obesity were allocated to case group; (3) Acyl or des-acyl ghrelin levels were measured after an overnight fasting (with or without postprandial concentrations); (4) Specific weight loss interventions on obesity, such as drugs, surgeries, regular exercises were disallowed before the measurement; (5) People included in these studies were in a relatively healthy condition, without genetic disorders known to cause obesity, eating disorders, heart disease, cancer, severe hepatic or renal disease, pregnancy, confirmed diagnosis of diabetes mellitus, uncontrolled hypertension, et al. To ensure maximum coverage of eligible studies, obese patients with metabolic syndrome (MetS, which was defined as the presence of three or more of following diagnostic criteria: abdominal obesity and waist circumference ≥ 88 cm for women or ≥ 102 cm for men; fasting plasma glucose > 6.1 mmol/L; circulating triglycerides ≥ 1.7 mmol/L; high-density lipoprotein < 40 mg/dl in men or < 50 mg/dl in women; hypertension including systolic pressure ≥ 140 mmHg or diastolic pressure ≥ 90 mmHg or antihypertensive treatment, according to the criteria of the National Cholesterol Education Program Adult Treatment Panel III guidelines)^50,51^ were included; (6) More than 6 points of Newcastle–Ottawa Scale (NOS)^52^ score were considered eligible for inclusion.

### Exclusion criteria

(1) Studies that only measured total ghrelin and failed to assess acyl and des-acyl ghrelin levels independently; (2) Abstracts, case reports, reviews or nonclinical studies; (3) Studies that were not written in English; (4) Studies lacking a healthy weight control group; (5) Studies that had duplicate data or repeat analysis; (6) The sample size of original articles was less than 10; (7) The data not presented as or could not be converted to the form of mean ± standard deviation (SD).

### Quality assessment and data collection

Quality assessment of the included articles was performed according to The Newcastle–Ottawa Quality Assessment Scale (NOS), which was designed to target nonrandomized studies and contains three different types of biases: bias of selection (0–4), bias of comparability (0–2) and bias of exposure (0–3). Studies with more than 6 points on the NOS score were considered eligible for inclusion^52^. A pretested standardized form was used to extract data from the included studies for study evaluation and evidence synthesis. The descriptive details included authors, population, sample size, sex, sample age, blood sample, handling methods, measuring methods, types of test meals, fasting and postprandial ghrelin levels. Both quality assessment and data extraction were also conducted independently by two reviewer authors (YM Wang and QX Wu), and discrepancies were identified and resolved through discussion with a third author (Q Chen).

### Statistical considerations

Stata/SE 15.0 for Mac (Stata Corp, College Station, TX, USA) was used to analyze the statistical data. The fasting and postprandial AG or DAG levels were summarized for each study sample and reported as the mean and the standard deviation (SD). Data presented as standard error (SE) were converted to SD by the equation SD = ${\text{SE}} \times \sqrt {{\text{number}}\;{\text{of}}\;{\text{subjects}}}$; moreover, when median and interquartile range appeared, a validated procedure was adopted to convert^53^ before being entered. Plasma DAG was calculated by subtracting AG from total ghrelin (TG)^54,55^ when studies happened to report AG and TG alone. As needed, data were obtained from graphs using Engauge Digitizer 12.1. The postprandial time points were chosen for consistency across the study protocols to allow for comparison. The changes in hormone concentrations from baseline to postprandial states were calculated as follows^56^: mean difference = ${\text{mean at postprandial}} - {\text{mean at baseline}}$, standard deviation of mean difference = $\sqrt {{\text{SD}}_{{{\text{baseline}}}}^{2} + {\text{SD}}_{{{\text{postprandial}}}}^{2} {-}2 \times {\text{r}} \times {\text{SD}}_{{{\text{baseline}}}} \times {\text{SD}}_{{{\text{postprandial}}}}}$, considering a correlation coefficient (r) of 0.5. When multiple relevant groups existed, formulas in the Cochrane Handbook were used to calculate the combined mean and SD^57^. Due to the different measuring methods with various units for ghrelin, continuous variables were expressed as standardized mean differences (SMDs) with 95% confidence intervals (CIs). *P*_SMD_ < 0.05 for any test or model was considered statistically significant. The I^2^ statistic and Cochrane’s Q test were measured to analyze the heterogeneity, and the cutoff values were 50% and 0.05, respectively. A fixed-effect model was used for the meta-analysis with moderate heterogeneity (I^2^ < 50%, *P*_heterogeneity_ > 0.05); otherwise, a random-effects model was performed, and a Galbraith plot was used to detect potential sources of heterogeneity. Subgroup analyses were performed according to blood sample handling and measuring methods. Publication bias was inspected by Begg’s funnel plots and Egger’s linear regression test when more than ten studies were involved, and a *P* value < 0.05 indicated potential publication bias.

## Results

### Study selection

**Figure 1:**
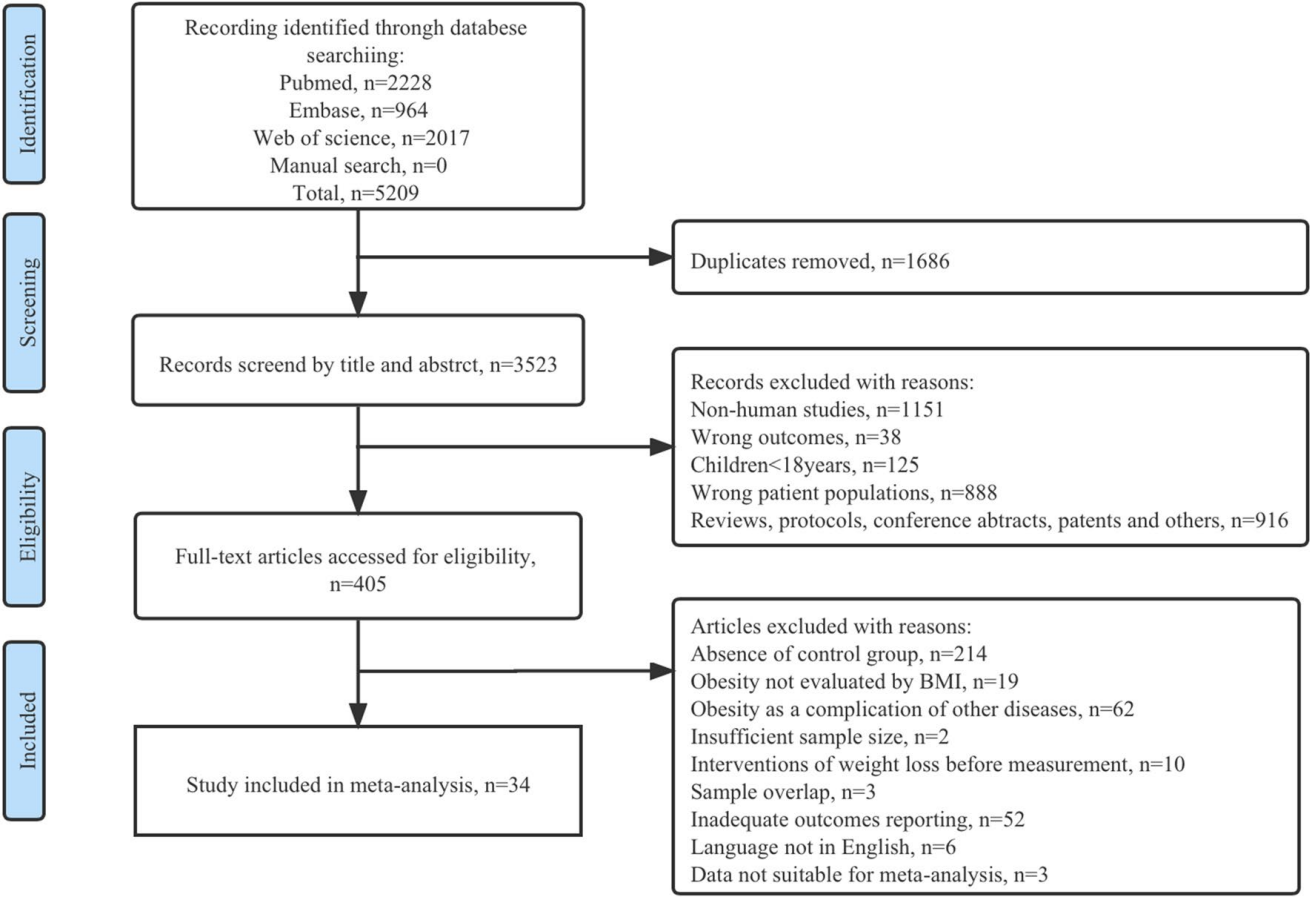
Flow diagram of the literature search and study selection process.

The PRISMA statement flow diagram outlines the procedures of literature identification, screening and study exclusion (Fig. 1). A total of 5209 putative articles were initially retrieved. After the removal of duplicates, reviewing titles and abstracts, and reading through full texts, 34 eligible articles^37–44,58–83^ that met the selection criteria were included in our systematic review and meta-analysis. The quality assessment of these studies is presented in Supplementary Fig. 1. All of the included studies had an NOS score over 6 points, which was considered high-quality.

### General characteristics

The general characteristics of the included studies are described in Table 1. An aggregate of 1863 adult participants (1125 obese patients and 738 healthy controls) were investigated in the 34 included trials. The mean participant BMI of each study ranged from 27.4 to 49.4 kg/m^2^ for patients and 18.5 to 23.2 kg/m^2^ for healthy controls. One Singapore^44^ study defined obesity with a BMI above 27.5 kg/m^2^ and healthy lean subjects with a BMI ranging from 18.5 to 23 kg/m^2^. For the remaining articles, BMI definition was based on the CDC criteria, and 6 articles^38,61,65,76,78,79^ included both obese patients and overweight subjects. All included participants had no genetic diseases identified as the cause of their obesity, and diabetes mellitus was considered an exclusion criterion, but obesity with MetS^40,64,74,80,83^ or obesity with morbidity^64,79,81^ and hypertension or hyperlipidemia under control by drugs^69,82^ were included.

**Table 1:** Summary of general characteristics of the included studies.

References	Country	Group	Sample size (male/female)	Age (year)	BMI (kg/m^2^)	Meal test	Out-come	Sampling time	Sample origin	Sample procedure	Technique
Baranowska^62^	Poland	Control	0/45	26.0 ± 7.6	21.5 ± 0.3	NA	AG	Fasting	Plasma	NR	RIA
		Obese	0/37	41.6 ± 12.4	32.7 ± 0.8						
Homaee^39^	Iran	Control	19/0	26.9 ± 1.3	18.5 ± 0.5	NA	AG	Fasting	Plasma	EDTA, Aprotinine, HCL	ELISA
		Obese	19/0	27.5 ± 1.3	31.0 ± 0.8						
Iceta^75^	France	Control	0/29	37.0 ± 2.0	21.5 ± 0.4	NA	AG, DAG	Fasting	Plasma	Parahydroxymercuribenzoic acid, HCL	ELISA
		Obese	0/55	38.0 ± 1.5	41.5 ± 0.8						
Kołodziejski^43^	Poland	Control	0/15	42.9 ± 5.3	22.3 ± 0.5	NA	AG, TG	Fasting	Serum	PMSF, HCL	RIA
		Obese	0/15	42.2 ± 3.3	39.8 ± 1.0						
Nakahara^65^	Japan	Control	0/11	25.7 ± 6.7	21.8 ± 3.1	NA	AG, DAG	Fasting	Plasma	EDTA, Aprotinine, HCL	ELISA
		Obese	0/10	27.7 ± 8.2	28.4 ± 2.7						
Ezquerro^74^	Spain	Control	11/19	44.0 ± 2.0	23.1 ± 0.5	NA	AG, DAG	Fasting	Plasma	NR	ELISA
		Obese-NG	16/28	39.0 ± 2.0	46.4 ± 1.3						
		Obese-IGT	17/25	44.0 ± 2.0	43.2 ± 1.0						
Haluzíková^69^	Czech Republic	Control	0/15	44.1 ± 2.8	22.2 ± 0.5	NA	AG	Fasting	Serum	DPP-IV inhibitor, Aprotinine	MILLIPLEX MAP
		Obese	0/17	39.9 ± 2.0	43.2 ± 1.7						
Tamboli^42^	USA	Control	0/9	36.0 ± 4.0	22.0 ± 1.0	NA	AG, TG	Fasting	Plasma	ETDA, Aprotinine, HCl	ELISA
		Obese	0/9	41.0 ± 4.0	44.0 ± 2.0						
Savage^71^	USA	Control	0/8	44.2 ± 5.5	22.7 ± 1.7	NA	AG	Fasting	Plasma	Aprotinine, HCL	RIA
		Obese	0/19	36.5 ± 1.5	38.6 ± 1.3						
Arafat^68^	Germany	Control	6/7	25.1 ± 0.6	21.7 ± 0.6	NA	AG, TG	Fasting	Plasma	NR	RIA
		Obese	5/6	28.4 ± 2.6	34.4 ± 1.7						
Rodríguez^40^	Spain	Control	25/30	56.0 ± 2.0	23.1 ± 0.3	NA	AG, DAG	Fasting	Plasma	NR	ELISA
		Obese-NG	41/25	55.0 ± 1.0	32.5 ± 0.5						
		Obese-IGT	21/16	59.0 ± 1.0	33.5 ± 0.8						
Dunn^67^	USA	Control	0/8	40.0 ± 3.2	23.0 ± 0.7	NA	AG	Fasting	Plasma	Aprotinine, HCL	RIA
		Obese	0/14	40.0 ± 2.1	40.0 ± 1.3						
Carroll^66^	USA	Control	5/12	NR	22.3 ± 0.5	NA	AG	Fasting	Plasma	EDTA, Aprotinine, DPP-IV inhibitor	RIA
		Obese	12/22	NR	43.4 ± 0.9						
Marzullo^58^	Italy	Control	10/10	31.7 ± 1.3	22.4 ± 0.6	NA	AG, TG	Fasting	Plasma	EDTA, HCL	RIA
		Obese	10/10	32.4 ± 1.6	41.3 ± 1.1						
Suematsu^61^	Japan	Control	16/1	36.0 ± 1.9	22.2 ± 0.6	NA	AG	Fasting	Plasma	NR	RIA
		Obese	16/1	35.5 ± 1.8	28.7 ± 1.2						
Bik^63^	Poland	Control	0/45	26.0 ± 7.6	21.5 ± 0.3	NA	AG	Fasting	Plasma	Aprotinine	RIA
		Obese	0/37	31.6 ± 8.2	32.7 ± 0.8						
Marzullo^37^	Italy	Control	8	NR	22.1 ± 1.2	NA	AG, TG	Fasting	Plasma	NR	RIA
		Obese	8	NR	33.7 ± 1.5						
Yunker^76^	USA	Control	25	NR	NR	NA	AG	Fasting	Plasma	NR	MILLIPLEX MAP
		Over weight	24	NR	NR						
		Obese	20	NR	NR						
Nogueira^80^	France	Control	5/16	33.0 ± 1.3	22.1 ± 0.6	NA	DAG	Fasting	Serum	NR	ELISA
		Obese-low HDL-c	6/15	34.0 ± 2.0	48.4 ± 1.8						
		Obese-MetS	6/11	38.0 ± 2.7	43.3 ± 1.1						
		Obese	4/17	37.0 ± 1.1	41.4 ± 0.9						
Lopez-Aguilar^77^	Mexico	Control	24/26	26.4 ± 0.8	22.7 ± 0.2	NA	AG	Fasting	Serum	Parahydroxymercuribenzoic acid, EDTA,	ELISA
		Obese	26/54	29.2 ± 0.70	35.4 ± 0.6						
Ozkan^79^	Turkey	Low weight	16/15	28.8 ± 2.9	17.6 ± 0.1	NA	AG	Fasting	Serum	Aprotinine	ELISA
		Normal weight	14/14	40.8 ± 3.9	21.7 ± 0.3						
		Over weight	15/15	52.2 ± 2.3	27.4 ± 0.4						
		Obese	16/15	52.1 ± 2.8	34.9 ± 0.4						
		Morbidly obese	15/15	45.8 ± 2.3	44.8 ± 0.8						
Gelisgen^81^	USA	Control	7/9	33.0 ± 1.6	23.0 ± 1.7	NA	AG	Fasting	Plasma	EDTA, Aprotinine,	ELISA
		Morbidly obese	9/12	35.2 ± 1.6	49.4 ± 5.3						
Karcz-Socha^82^		Control	22/24	51.2 ± 1.0	23.4 ± 0.2	NA	AG, TG	Fasting	Plasma	EDTA, Aprotinine, DPP-IV inhibito, PMSF	RIA
		Moderately obese	21/22	50.5 ± 0.9	32.5 ± 0.2						
		Morbidly obese	27/26	52.3 ± 0.9	37.5 ± 0.2						
Krzyzanowska-Swiniarska^83^	Poland	Control	0/32	28.8 ± 0.8	21.3 ± 0.3	NA	AG	Fasting	Serum	Aprotinine, HCL, PMSF	RIA
		Obese without insulin resistance	0/30	32.5 ± 1.2	34.4 ± 0.7						
		Obese with insulin resistance	0/30	32.3 ± 1.2	37.8 ± 1.0						
Zwirska-Korczala^64^	Poland	Control	0/8	33.9 ± 3.7	23.2 ± 0.7	Standard mixed breakfast (527 kcal)	AG,TG	Fasting and postprandial 30, 60, 120 min	Plasma	EDTA, PMSF, HCL, DPP-IV inhibitor	RIA
		Moderately obese-MetS	0/12	37.1 ± 2.2	34.9 ± 0.9						
		Morbidly obese-MetS	0/17	32.3 ± 1.7	46.9 ± 1.6						
Rizi^44^	Singapore	Control	9/0	23.2 ± 0.2	22 ± 0.2	High-protein test, high-fat test, high-carbohydrate test (isocaloric 600 kcal)	AG	Fasting and postprandial 30, 60, 90, 120, 180 min	Plasma	EDTA, DPP-IV inhibitor, Aprotinine	ELISA
		Obese	9/0	28.6 ± 1.4	30.1 ± 0.7						
Brede^73^	Germany	Control	20/0	24.1 ± 3.7	22.4 ± 1.5	Ad libitum test buffet (1500 kcal)	AG,TG	Fasting and postprandial 30 minites	Plasma	Aprotinine	RIA
		Obese	20/0	25.2 ± 3.7	34.9 ± 3.6						
Douglas^78^	UK	Control	10/10	37.5 ± 3.4	22.4 ± 0.3	Standard breakfast (643 kcal for males and 513 kcal for females)	AG, DAG	Fasting and postprandial 30, 60, 90 min	Plasma	Aprotinine	ELISA
		Obese	12/11	45 ± 2.6	29.2 ± 0.6						
Seyssel^72^	Spain	Control	20/0	27.0 ± 1.0	22.0 ± 0.3	Standard mixed breakfast (706 kcal)	AG	Fasting and postprandial 60 minites	Plasma	Parahydroxymercuribenzoic acid, EDTA, HCl	ELISA
		Obese	17/0	29.0 ± 2.0	31.9 ± 0.4						
Dardzińska^41^	Poland	Control	1/12	37.2 ± 2.6	23.0 ± 1.0	Mixed-meal (300 kcal)	AG, DAG	Fasting and postprandial 120 minites	Plasma	EDTA, Parahydroxymercuribenzoic acid	ELISA
		Obese	7/17	35.4 ± 1.9	43.8 ± 0.7						
Heden^70^	USA	Control	14	26 ± 1.6	22.9 ± 0.5	Mixed meal (600 kcal)	AG	Fasting and postprandial 5, 10, 15, 20, 30, 40, 50, 60, 75, 90, 120, 150, 180, 210, 240 min	Plasma	EDTA, Aprotinine	MILLIPLEX MAP
		Obese	14	25.1 ± 1.3	34.8 ± 1.2						
Ueda^38^	Japan	Control	7/0	22.4 ± 1.6	22.4 ± 0.9	Standard breakfast (560 kcal)	AG	Fasting and postprandial 60, 90, 120, 150, 180 min	Plasma	EDTA, Aprotinine	ELISA
		Obese	7/0	22.9 ± 1.3	30 ± 1.2						
Tentolouris^59^	Greece	Control	0/8	40.2 ± 4.0	21.8 ± 0.8	Carbohydrate-rich meal (546 kcal), fat-rich meal (532 kcal)	AG	Fasting and postprandial 60, 120, 180 min	Plasma	NR	RIA
		Obese	0/8	39.9 ± 5.3	35.51 ± 1.6						
Foschi^60^	Italy	Control	3/3	26.2 ± 0.7	22.5 ± 0.7	Liquid test meal (504 kcal)	AG	Fasting and postprandial 60, 120, 180 min	Plasma	EDTA	RIA
		Obese	1/11	41.1 ± 3.8	42.9 ± 1.3						

Age and BMI are presented as the means ± SEM; *MetS* metabolic syndrome, *HDL-c* high-density lipoprotein-cholesterol, *NG* normoglycemia, *IGT* impaired glucose tolerance, *AG* acyl ghrelin, *DAG* des-acyl ghrelin, *ELISA* enzyme-linked immunosorbent assay, *RIA* radioimmunoassay, *MILLIPLEX MAP* magnetic bead-based quantitative multiplex immunoassay, *EDTA* ethylene diamine tetraacetic acid, *PMSF* phenylmethylsulfonyl fluoride, *HCL* hydrogen chloride, *NA* not applicable, *NR* not reported.

### Fasting AG

**Figure 2:**
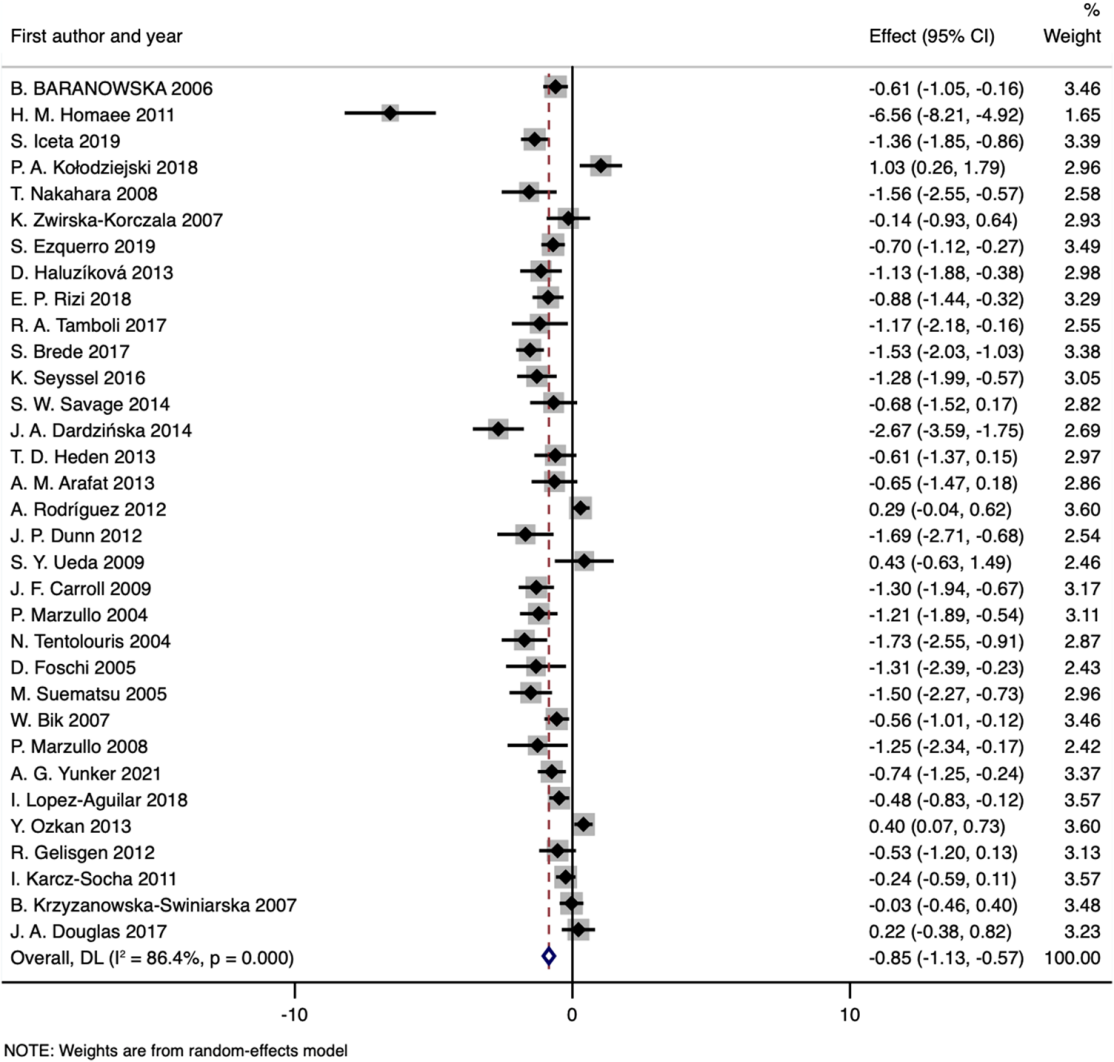
Forest plot for comparisons of fasting AG levels (obesity vs. normal weight).

Thirty-three included studies^37–44,58–79,81–83^ with 1066 obese cases and 717 healthy controls measured baseline fasting circulating acyl ghrelin levels in obese patients and control subjects. The pooled effect size showed significantly lower levels of baseline AG in the obese groups than in the healthy controls (SMD: − 0.85; 95% CI: − 1.13 to − 0.57; *P*_SMD_ < 0.001) (Fig. 2 and Table 2). Interstudy heterogeneity was significant, with an I^2^ of 86.4% (*P*_heterogeneity_ < 0.001), and a random-effects model was applied. Ten studies^38–41,43,73,78,79,82,83^ were identified as the main contributors to heterogeneity by using Galbraith plots (Supplementary Fig. 2). The heterogeneity was effectively decreased after excluding the outlier comparisons, and the SMD value and 95% CI did not change substantially (SMD obtained from fixed-effects model: − 0.86; 95% CI: − 0.99 to − 0.73; I^2^:36.6%; *P*_heterogeneity_ = 0.041). Similar results were observed when subgroup analyses stratified by the assay technique or storage procedure showed a robust decrease in fasting AG levels of obese patients for each subgroup, and the exclusion of outlier studies did not change the significance of the results (Table 2).

**Table 2:** Meta-analysis for comparison of fasting AG levels (obesity vs. normal weight).

Groups or subgroups	N	References	Random-effects model		Fix-effects model		I^2^ (%)	*P*_Heterogeneity_
			SMD (95%CI)	*P*_SMD_	SMD (95%CI)	*P*_SMD_		
**Fasting AG**								
All	33	^37–44,58–79,81–83^	− 0.85 (− 1.13 to − 0.57)	< 0.001	− 0.55 (− 0.65 to − 0.45)	< 0.001	86.4	< 0.001
**Subgroup 1 technique**								
ELISA	14	^38–42,44,65,72,74,75,77–79,81^	− 0.95 (− 1.47 to − 0.43)	< 0.001	− 0.41 (− 0.55 to − 0.27)	< 0.001	91.7	< 0.001
RIA	16	^37,58–64,66–68,71,82,83^	− 0.80 (− 1.14 to − 0.45)	< 0.001	− 0.66 (− 0.81 to − 0.51)	< 0.001	78.5	< 0.001
MILLIPLEX MAP	3	^69,70,76^	− 0.81 (− 1.17 to − 0.44)	< 0.001	− 0.81 (− 1.17 to − 0.44)	< 0.001	0.0	0.596
**Subgroup 2 enzymatic inhibitors contained**								
YES	25	^37–40,58–74,76–79,81–83^	− 0.87 (− 1.22 to − 0.53)	< 0.001	− 0.57 (− 0.68 to − 0.46)	< 0.001	87.5	< 0.001
NO	8	^37,40,59,61,62,68,74,76^	− 0.79 (− 1.28 to − 0.31)	0.001	− 0.49 (− 0.67 to − 0.30)	< 0.001	83.1	< 0.001
**Fasting AG after excluding the studies with heterogeneity**								
All	23	^37,42,44,58–72,74–77,81^	− 0.92 (− 1.09 to − 0.75)	< 0.001	− 0.86 (− 0.99 to − 0.73)	< 0.001	36.6	0.041
**Subgroup 1 technique**								
ELISA	8	^42,44,65,72,74,75,77,81^	− 0.91 (− 1.19 to − 0.62)	< 0.001	− 0.83 (− 1.03 to − 0.64)	< 0.001	47.6	0.064
RIA	12	^37,58–64,66–68,71^	− 0.98 (− 1.27 to − 0.70)	< 0.001	− 0.91 (− 1.11 to − 0.71)	< 0.001	44.9	0.046
MILLIPLEX MAP	3	^69,70,76^	− 0.81 (− 1.17 to − 0.44)	< 0.001	− 0.81 (− 1.17 to − 0.44)	< 0.001	0.0	0.596
**Subgroup 2 enzymatic inhibitors contained**								
YES	16	^42,44,58,60,63–67,69–72,75,77,81^	− 0.93 (− 1.15 to − 0.71)	< 0.001	− 0.87 (− 1.03 to − 0.71)	< 0.001	40.0	0.050
NO	7	^37,59,61,62,68,74,76^	− 0.91 (− 1.22 to − 0.61)	< 0.001	− 0.85 (− 1.07 to − 0.62)	< 0.001	38.6	0.134

*AG* acyl ghrelin, *ELISA* enzyme-linked-immunosorbent-assay, *RIA* radio-immuno-assay, *MILLIPLEX MAP* magnetic bead-based quantitative multiplex immunoassay, *N* number of studies.

### Fasting DAG

**Figure 3:**
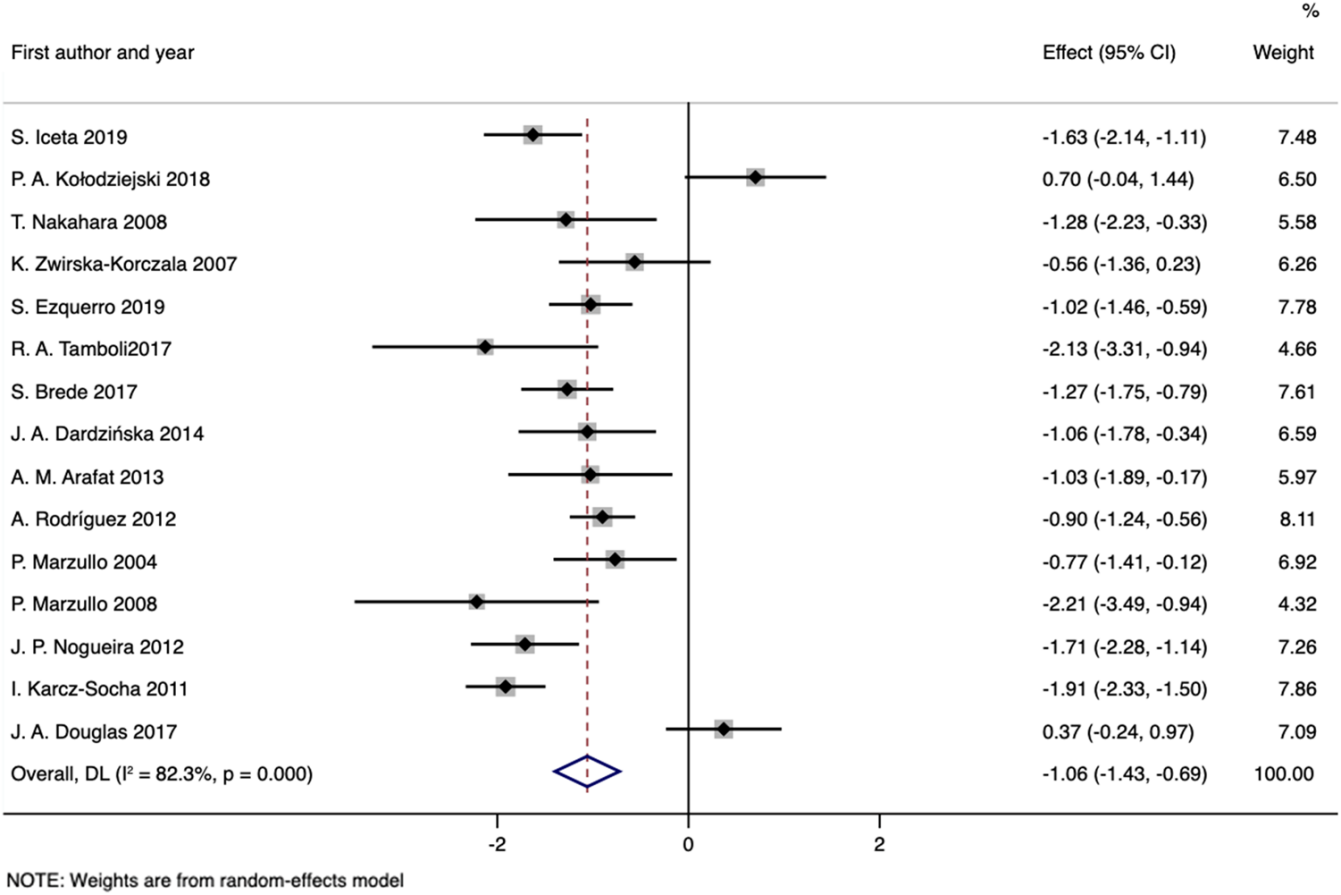
Forest plot for comparisons of fasting DAG levels (obesity vs. normal weight).

The fasting circulation concentrations of des-acyl ghrelin were reported in 7 studies^40,41,65,74,75,78,80^ with 360 obese patients and 179 healthy controls; meanwhile, 8 studies^37,42,43,58,64,68,73,82^ with 208 patients and 139 controls measured both blood acyl ghrelin and total ghrelin baseline levels, which were used to calculate the DAG levels. Pooled analysis showed that circulating fasting DAG levels were significantly decreased in obese patients compared with control subjects (SMD obtained from random-effects model: − 1.06; 95% CI: − 1.43 to − 0.69; *P*_SMD_ < 0.001), although the overall heterogeneity was apparent (I^2^: 82.3%, *P*_heterogeneity_ < 0.001) (Fig. 3 and Table 3). The Galbraith plot indicated that the assay results of 4 articles^43,78,80,82^ were largely responsible for this heterogeneity (Supplementary Fig. 3). Exclusion of these studies resulted in an SMD of − 1.11 (− 1.29 to − 0.94; *P*_SMD_ < 0.001; fixed-effects model) with a significant decrease in heterogeneity (I^2^: 32.0%; *P*_heterogeneity_ = 0.143). The results of our meta-analyses were also consistent in subgroup analyses regardless of outlier study inclusion or exclusion (Table 3).

**Table 3:** Meta-analysis for comparison of fasting DAG levels (obesity vs. normal weight).

Groups or subgroups	N	References	Random-effects model		Fix-effects model		I^2^ (%)	*P*_Heterogeneity_
			SMD (95%CI)	*P*_SMD_	SMD (95%CI)	*P*_SMD_		
**Fasting DAG**								
All	15	^37,40–43,58,64,65,68,73–75,78,80,82^	− 1.06 (− 1.43 to − 0.69)	< 0.001	− 1.09 (− 1.24 to − 0.94)	< 0.001	82.3	< 0.001
**Subgroup 1 technique**								
ELISA	8	^40–42,65,74,75,78,80^	− 1.12 (− 1.58 to − 0.65)	< 0.001	− 1.16 (− 1.39 to − 0.92)	< 0.001	86.2	< 0.001
RIA	7	^37,43,58,64,68,73,82^	− 0.98 (− 1.66 to − 0.31)	0.004	− 1.05 (− 1.24 to − 0.86)	< 0.001	80.2	< 0.001
**Subgroup 2 enzymatic inhibitors contained**								
YES	10	^41–43,58,64,65,73,75,78,82^	− 0.94 (− 1.51 to − 0.37)	0.001	− 1.07 (− 1.27 to − 0.88)	< 0.001	87.2	< 0.001
NO	5	^37,40,68,74,80^	− 1.22 (− 1.61 to − 0.84)	< 0.001	− 1.12 (− 1.35 to − 0.89)	< 0.001	54.7	0.066
**Fasting DAG after excluding the studies with heterogeneity**								
All	11	^37,40–42,58,64,65,68,73–75^	− 1.14 (− 1.38 to − 0.91)	< 0.001	− 1.11 (− 1.29 to − 0.94)	< 0.001	32.0	0.143
**Subgroup 1 technique**								
ELISA	6	^40–42,65,74,75^	− 1.20 (− 1.51 to − 0.89)	< 0.001	− 1.14 (− 1.35 to − 0.92)	< 0.001	40.6	0.135
RIA	5	^37,58,64,68,73^	− 1.06 (− 1.47 to − 0.65)	< 0.001	− 1.07 (− 1.38 to − 0.76)	< 0.001	35.1	0.187
**Subgroup 2 enzymatic inhibitors contained**								
YES	7	^41,42,58,64,65,73,75^	− 1.21 (− 1.53 to − 0.88)	< 0.001	− 1.22 (− 1.47 to − 0.97)	< 0.001	36.5	0.150
NO	4	^37,40,68,74^	− 1.03 (− 1.35 to − 0.72)	< 0.001	− 1.00 (− 1.26 to − 0.75)	< 0.001	21.3	0.282

*DAG* des-acyl ghrelin, *ELISA* enzyme-linked-immunosorbent-assay, *RIA* radio-immuno-assay, *MILLIPLEX MAP* magnetic bead-based quantitative multiplex immunoassay, *N* number of studies.

### Postprandial AG

**Figure 4:**
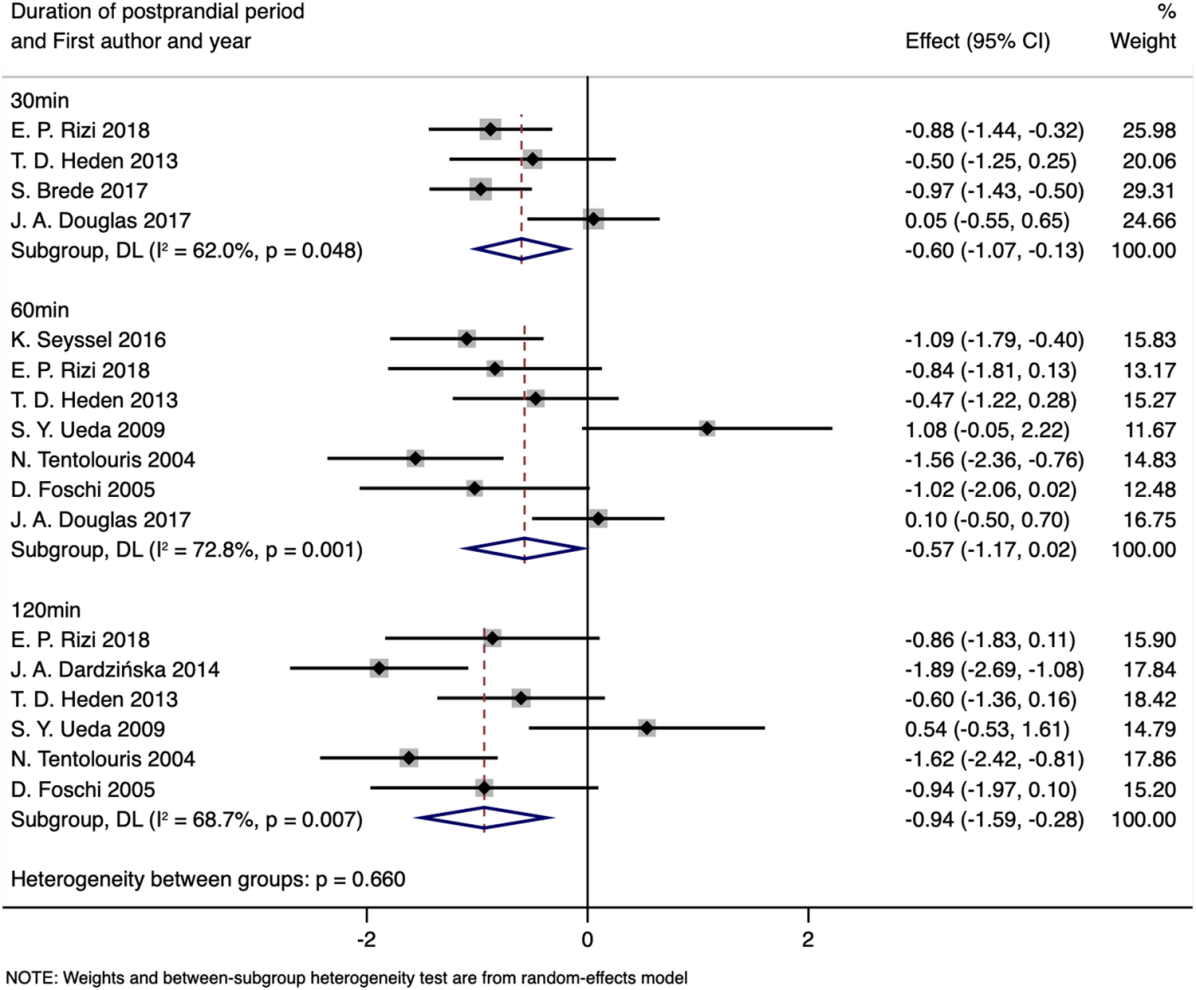
Forest plot for comparisons of postprandial AG levels stratified by duration of postprandial period (obesity vs. normal weight).

Ten articles^38,41,44,59,60,64,70,72,73,78^ presented the data on postprandial concentrations of acyl ghrelin, although different measurement times were reported. According to the number of studies within each time stratification point, we selected 30, 60 and 120 min to conduct the meta-analyses. After exclusion of the study by Zwirska-Korczala et al.^64^ due to the lack of statistical data of standard deviation and the other two articles^38,78^ due to high heterogeneity (Supplementary Fig. 4), 3, 5 and 5 studies were included to compare the circulating AG levels between obese subjects and controls at 30, 60 and 120 min postprandial, respectively (Table 4). Stratified analyses demonstrated significantly lower levels of postprandial AG in obese subjects at each time point with a fixed-effects model. The SMD values and 95% CIs of 30 min postprandial (SMD: − 0.85; 95% CI: − 1.18 to − 0.53; *P*_SMD_ < 0.001; I^2^:0.0%, *P*_heterogeneity_ = 0.577), 60 min postprandial (SMD: − 1.00; 95% CI: − 1.37 to − 0.63; *P*_SMD_ < 0.001; I^2^: 0.0%, *P*_heterogeneity_ = 0.410) and 120 min postprandial (SMD: − 1.21; 95% CI: − 1.59 to − 0.83; *P*_SMD_ < 0.001; I^2^: 42.2%, *P*_heterogeneity_ = 0.140) did not change substantially after excluding the outlier comparison (Fig. 4 and Table 4).

**Table 4:** Meta-analysis for comparison of postprandial AG levels stratified by duration of postprandial period (obesity vs. normal weight).

Groups or subgroups	N	References	Random-effects model		Fix-effects model			
			SMD (95%CI)	*P*_SMD_	SMD (95%CI)	*P*_SMD_	I^2^ (%)	*P*_Heterogeneity_
**Postprandial AG stratified by duration of postprandial period**								
30 min	4	^44,70,73,78^	− 0.60 (− 1.07 to − 0.13)	0.013	− 0.65 (− 0.93 to − 0.36)	< 0.001	62.0	0.048
60 min	7	^38,44,59,60,70,72,78^	− 0.57 (− 1.17 to 0.02)	0.026	− 0.57 (− 0.87 to − 0.27)	< 0.001	72.8	0.001
120 min	6	^38,41,44,59,60,70^	− 0.94 (− 1.59 to − 0.28)	0.005	− 1.01 (− 1.37 to − 0.65)	< 0.001	68.7	0.007
**Postprandial AG stratified by duration of postprandial period after excluding the studies with heterogeneity**								
30 min	3	^44,70,73^	− 0.85 (− 1.18 to − 0.53)	< 0.001	− 0.85 (− 1.18 to − 0.53)	< 0.001	0.0	0.577
60 min	5	^44,59,60,70,72^	− 1.00 (− 1.37 to − 0.63)	< 0.001	− 1.00 (− 1.37 to − 0.63)	< 0.001	0.0	0.410
120 min	5	^41,44,59,60,70^	− 1.20 (− 1.71 to − 0.69)	< 0.001	− 1.21 (− 1.59 to − 0.83)	< 0.001	42.2	0.140

*AG* acyl ghrelin.

### Changes in postprandial AG

**Figure 5:**
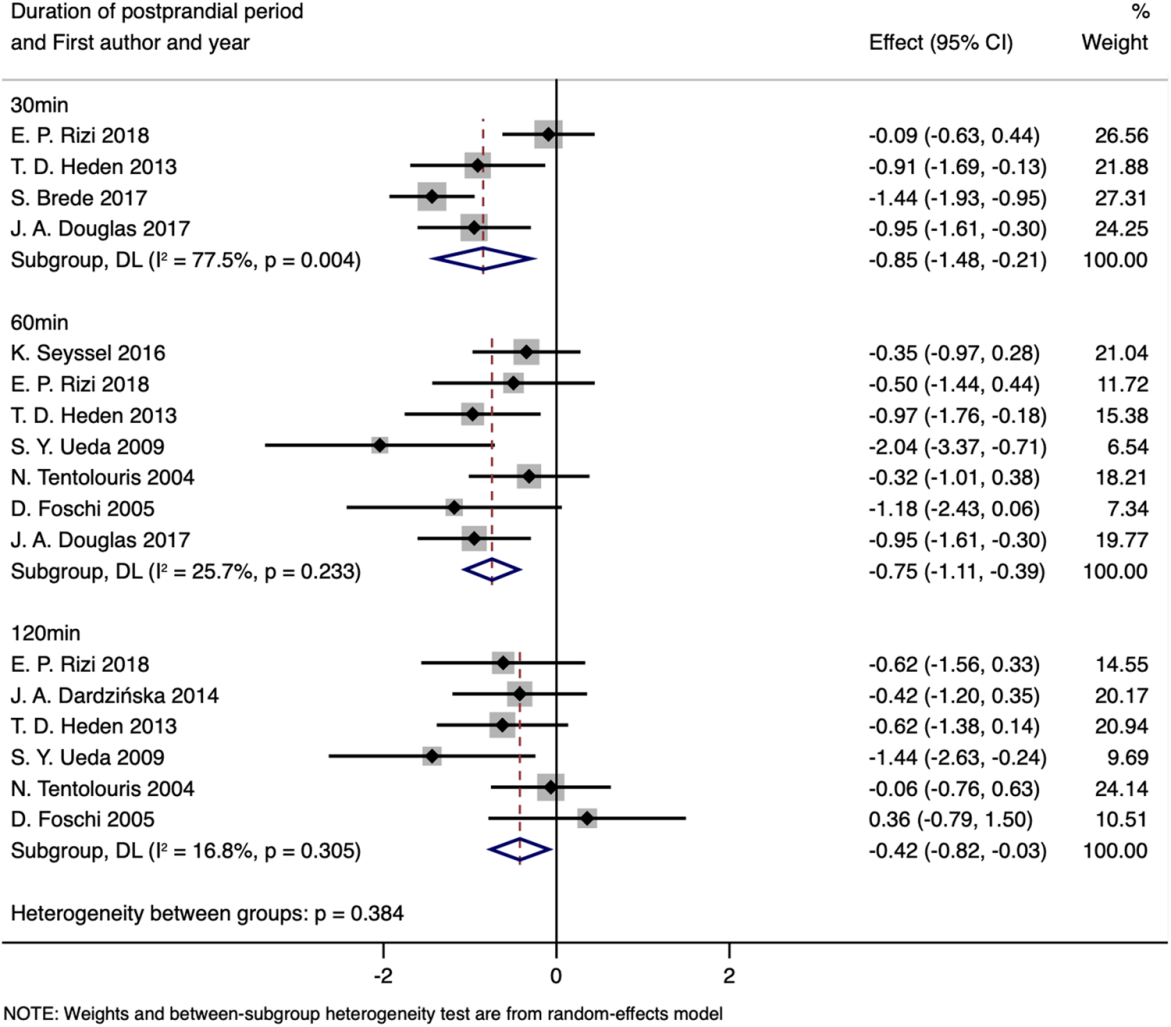
Forest plot for comparisons of postprandial AG levels stratified by duration of postprandial period in normal-weight group (postprandial vs. baseline).

For healthy individuals, stratification by time points demonstrated a statistically significant decrease in blood AG levels in the 30 min (SMD obtained from random-effects model: − 0.85; 95%CI: − 1.48 to − 0.21; *P*_SMD_ = 0.009; I^2^:77.5%; *P*_heterogeneity_ = 0.004), 60 min (SMD obtained from fixed-effects model: − 0.72; 95%CI: − 1.02 to − 0.41; *P*_SMD_ < 0.001; I^2^:25.7%; *P*_heterogeneity_ = 0.233) and 120 min (SMD obtained from fixed-effects model: − 0.42; 95%CI: − 0.77 to − 0.06; *P*_SMD_ = 0.021; I^2^:16.8%; *P*_heterogeneity_ = 0.305) following meal test intervention compared with fasting states (Fig. 5 and Table 5). A high level of heterogeneity can be observed in the stratification of 30 min, and the study by E. P. Rizi et al.^44^ was considered the main cause of the heterogeneity via the Galbraith plot (Supplementary Fig. 5). With the exclusion of this article, heterogeneity decreased significantly (I^2^:0.0%; *P*_heterogeneity_ = 0.371), and the significance of the result remained consistent (SMD obtained from the fixed-effects model: − 1.19; 95% CI: − 1.54 to − 0.84; *P*_SMD_ < 0.001).

**Table 5:** Meta-analysis for comparison of postprandial AG levels stratified by duration of postprandial period (postprandial vs. baseline).

Groups or subgroups	N	References	Random-effects model		Fix-effects model		I^2^ (%)	*P*_Heterogeneity_
			SMD (95%CI)	*P*_SMD_	SMD (95%CI)	*P*_SMD_		
**Postprandial AG stratified by duration of postprandial period in normal-weight group**								
30 min	4	^44,70,73,78^	− 0.85 (− 1.48 to − 0.21)	0.009	− 0.86 (− 1.15 to − 0.56)	< 0.001	77.5	0.004
60 min	7	^38,44,59,60,70,72,78^	− 0.75 (− 1.11 to − 0.39)	< 0.001	− 0.72 (− 1.02 to − 0.41)	< 0.001	25.7	0.233
120 min	6	^38,41,44,59,60,70^	− 0.42 (− 0.82 to − 0.03)	0.034	− 0.42 (− 0.77 to − 0.06)	0.021	16.8	0.305
**Postprandial AG stratified by duration of postprandial period in normal-weight group after excluding the studies with heterogeneity**								
30 min	3	^70,73,78^	− 1.19 (− 1.54 to − 0.84)	< 0.001	− 1.19 (− 1.54 to − 0.84)	< 0.001	0.0	0.371
60 min	7	^38,44,59,60,70,72,78^	− 0.75 (− 1.11 to − 0.39)	< 0.001	− 0.72 (− 1.02 to − 0.41)	< 0.001	25.7	0.233
120 min	6	^38,41,44,59,60,70^	− 0.42 (− 0.82 to − 0.03)	0.034	− 0.42 (− 0.77 to − 0.06)	0.021	16.8	0.305
**Postprandial AG stratified by duration of postprandial period in obese group**								
30 min	4	^44,70,73,78^	− 0.61 (− 0.89 to − 0.34)	< 0.001	− 0.61 (− 0.89 to − 0.34)	< 0.001	0.0	0.762
60 min	7	^38,44,59,60,70,72,78^	− 0.62 (− 0.94 to − 0.30)	< 0.001	− 0.62 (− 0.91 to − 0.33)	< 0.001	15.9	0.390
120 min	6	^38,41,44,59,60,70^	− 0.31 (− 0.68 to 0.05)	0.092	− 0.28 (− 0.60 to 0.03)	0.074	24.0	0.254

*AG* acyl ghrelin.

**Figure 6:**
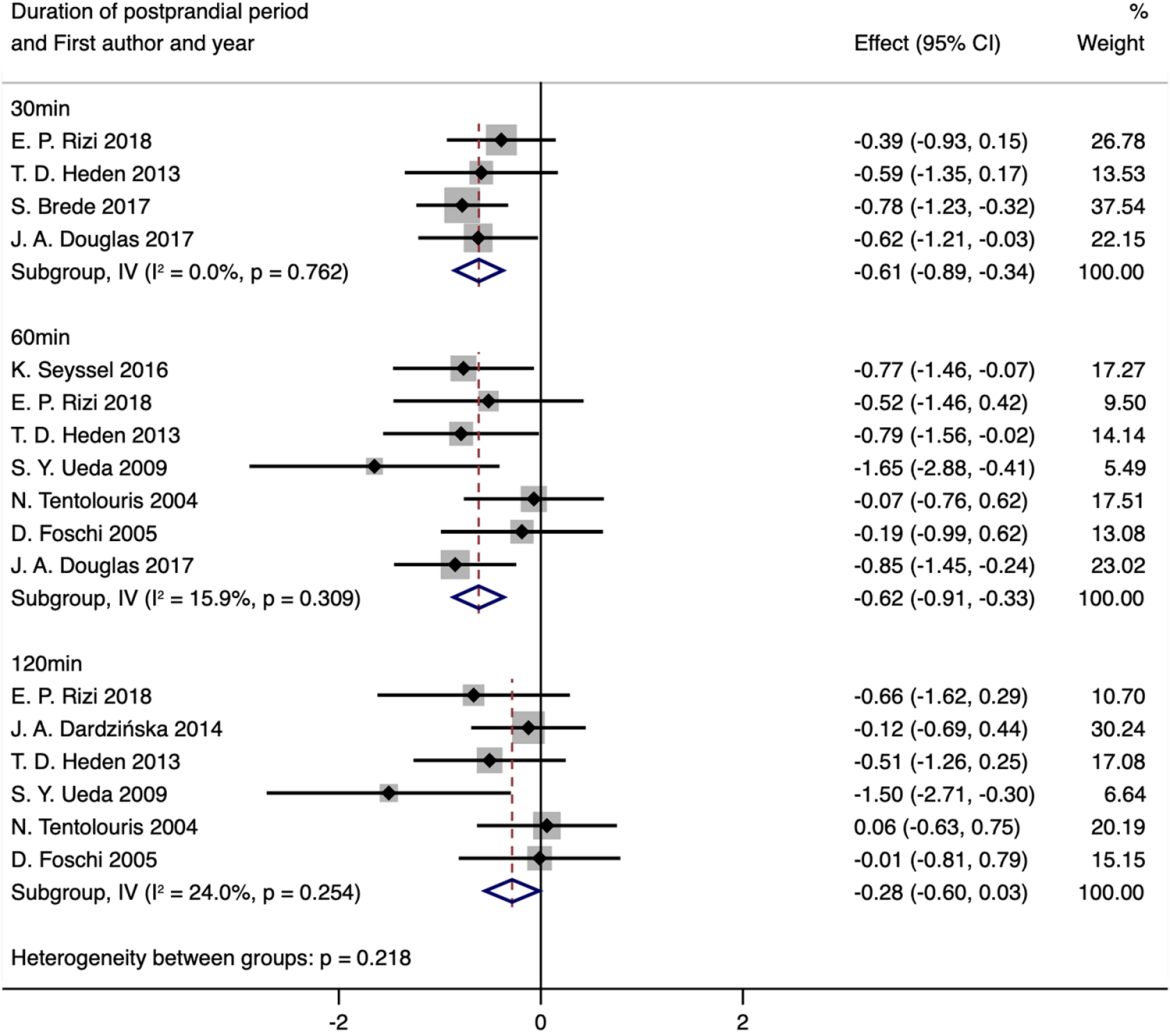
Forest plot for comparisons of postprandial AG levels stratified by duration of postprandial period in the obese group (postprandial vs. baseline).

For obese individuals, stratification by time points demonstrated a statistically significant decrease in blood AG levels in the 30 min (SMD obtained from fixed-effects model: − 0.61; 95% CI: − 0.89 to − 0.34; *P*_SMD_ < 0.001; I^2^:0.0%; *P*_heterogeneity_ = 0.762) and 60 min (SMD obtained from fixed-effects model: − 0.62; 95% CI: − 0.91 to − 0.33; *P*_SMD_ < 0.001; I^2^:15.9%; *P*_heterogeneity_ = 0.309) following meal test intervention, but there was no significant difference between postprandial 120 min and baseline states (SMD obtained from fixed-effects model: − 0.28; 95% CI: − 0.60 to 0.03; *P*_SMD_ = 0.074; I^2^:24.0%; *P*_heterogeneity_ = 0.254) (Fig. 6 and Table 5).

**Figure 7:**
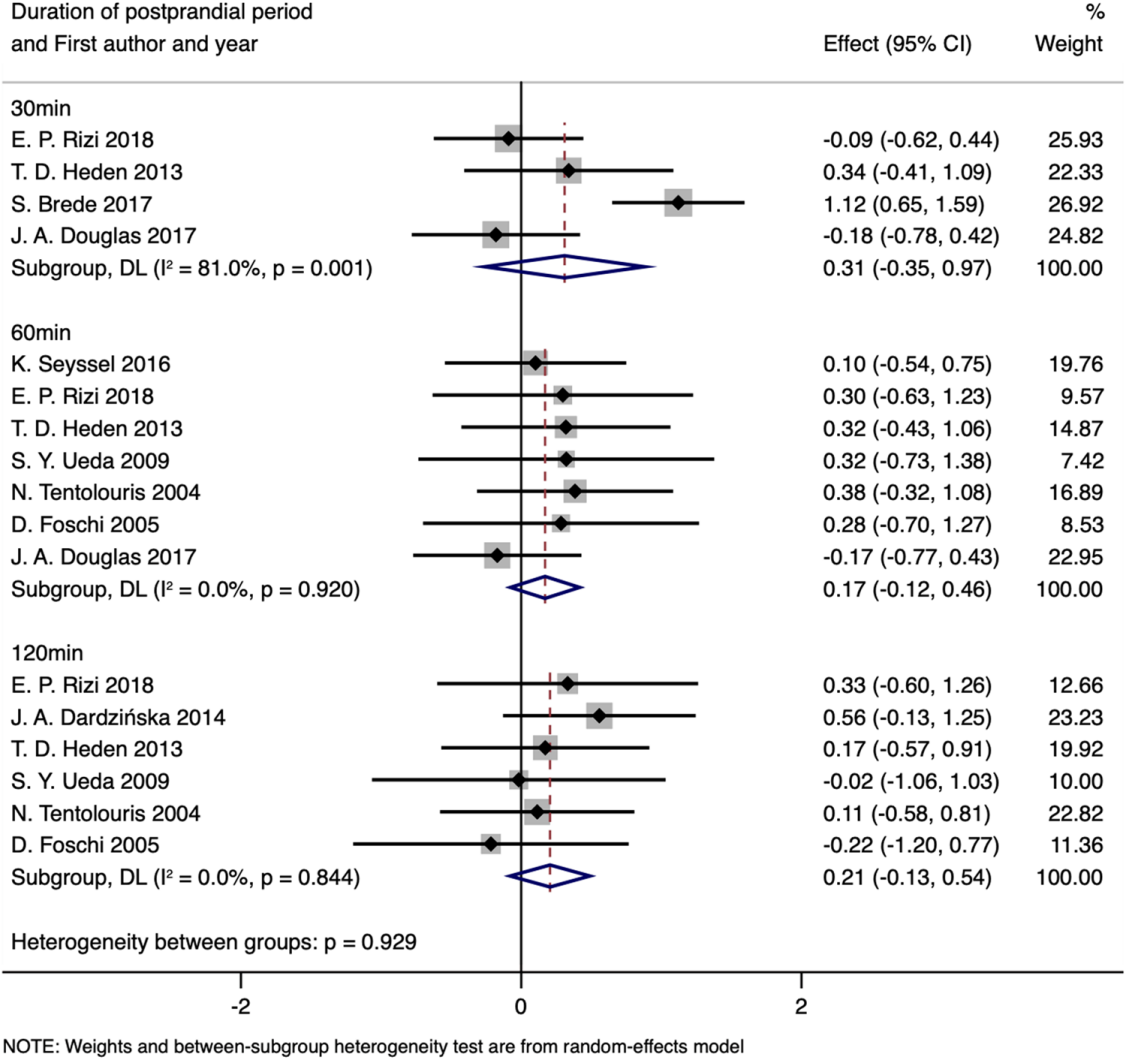
Forest plot of the changes in postprandial AG levels stratified by the duration of the postprandial period (obesity vs. normal weight).

The mean change of AG concentration (calculated as the differential between baseline and postprandial states) was similar in both obese and lean health groups at each time point (ΔSMD_30min_: 0.31, 95%CI: − 0.35 to 0.97, *P*_SMD_ = 0.359, I^2^:81.0%; *P*_heterogeneity_ = 0.001; ΔSMD_60min_: 0.17, 95%CI: − 0.12 to 0.46, *P*_SMD_ = 0.246, I^2^:0.0%; *P*_heterogeneity_ = 0.920; ΔSMD_120min_: 0.21, 95% CI: − 0.13 to 0.54, *P*_SMD_ = 0.224, I^2^:0.0%; *P*_heterogeneity_ = 0.884, random-effects model, Fig. 7 and Table 6), even the exclusion of the study by S. Brede et al.^73^ in the stratification of 30 min for heterogeneity (Supplementary Fig. 6), the results of our meta-analyses remained consistent (ΔSMD_30min_: 0.03, 95% CI: − 0.38 to 0.33, *P*_SMD_ = 0.887, I^2^:0.0%; *P*_heterogeneity_ = 0.541, Table 6).

**Table 6:** Meta-analysis of changes in postprandial AG levels stratified by duration of the postprandial period (obesity vs. normal weight).

Groups or subgroups	N	References	Random-effects model		Fix-effects model		I^2^ (%)	*P*_Heterogeneity_
			SMD (95%CI)	*P*_SMD_	SMD (95%CI)	*P*_SMD_		
**Change of postprandial AG stratified by duration of postprandial period**								
30 min	4	^44,70,73,78^	0.31 (− 0.35 to 0.97)	0.359	0.38 (0.10 to 0.67)	0.008	81.0	0.001
60 min	7	^38,44,59,60,70,72,78^	0.17 (− 0.12 to 0.46)	0.246	0.17 (− 0.12 to 0.46)	0.246	0.0	0.920
120 min	6	^38,41,44,59,60,70^	0.21 (− 0.13 to 0.54)	0.224	0.21 (− 0.13 to 0.54)	0.224	0.0	0.884
**Change of postprandial AG stratified by duration of postprandial period after excluding the studies with heterogeneity**								
30 min	3	^44,70,78^	0.03 (− 0.38 to 0.33)	0.887	0.03 (− 0.38 to 0.33)	0.887	0.0	0.541
60 min	7	^38,44,59,60,70,72,78^	0.17 (− 0.12 to 0.46)	0.246	0.17 (− 0.12 to 0.46)	0.246	0.0	0.920
120 min	6	^38,41,44,59,60,70^	0.21 (− 0.13 to 0.54)	0.224	0.21 (− 0.13 to 0.54)	0.224	0.0	0.844

*AG* acyl ghrelin.

### Postprandial DAG

Only four included studies^41,64,73,78^ reported postprandial TG or DAG levels, and one of them was excluded because of the lack of standard deviation data for the DAG calculation. The remaining three studies investigated peripheral blood hormones after the meal test, but time points were inconsistent and were not suitable for a meta-analysis, as such, this postprandial DAG group was not considered further.

### Publication bias

The results of Egger’s and Begg’s tests detected that there might be a publication bias for the outcome of fasting AG levels (Pr > |z| = 0.010 for Begg’s test and *P* > |t| = 0.000 for Egger’s test) (Supplementary Fig. 7). To clarify this problem, a trim-and-fill method was used to adjust the results, no trimming was performed, and the data were unchanged. There was no publication bias in the literature, and the significant *P* value of Begg’s and Egger’s tests may originate from other factors, such as mixed age, gender or ethnicity, in some studies. No publication bias was detected in the fasting DAG analysis (Pr > |z| = 0.843 for Begg's test and *P* > |t| = 0.792 for Egger's test) (Supplementary Fig. 8).

## Discussion

Ghrelin, an endogenous ligand of the GHSR, is the only known orexigenic gut hormone that increases appetite and food reward^11,45^. Although AG and DAG were described separately since ghrelin was first introduced in 1999 (Kojima et al.^11^), previous studies preferred to examine total plasma ghrelin without distinguishing AG and DAG before the theory that DAG may have independent actions suggested by Broglio et al. in 2004^22^. With the swiftly rising prevalence of obesity, it is important to evaluate appetite-related hormones precisely, the differences in which could both inform mechanisms in obesity and offer potential new pharmacological interventions.

To the best of our knowledge, this was the first systematic review and meta-analysis to compare the concentrations of AG and DAG separately between obese patients and healthy individuals while also considering the dietary states that can affect ghrelin levels. The main findings were that under a fasting state, both AG and DAG decreased significantly in obese groups compared with controls; for the postprandial state, a similar extent of AG decline can be observed in both groups, and a shorter duration of suppression existed in obese groups.

### Fasting acyl and des-acyl ghrelin in obesity

Several studies have reported that obese individuals have higher fasting levels of circulating acyl ghrelin than lean subjects^38,40,43^, indicating that AG may play a key role in the cause of obesity directly or indirectly via stimulation of food intake. However, according to this meta-analysis, we demonstrated a reduction in circulating basal AG levels in obese adults (Fig. 2 and Table 2). Significant heterogeneity did exist, and after excluding the outlier studies that were identified by the Galbraith plots, the significance of the result remained virtually unchanged (Table 2). Similar reductions were also observed in the obese patients when circulating fasting DAG levels were pooled (Fig. 3 and Table 3). The simultaneous variation of AG and DAG can be partially explained by the common sense that esterase-catalyzed deacylation produces DAG from AG, and after intravenous injection, AG appears to induce the secretion of DAG in humans^84^. In addition, the reacylation of DAG to AG by the catalysis of plasma membrane-exposed GOAT has been proposed^85^.

The significant drop in both AG and DAG supports the hypothesis of physically compensatory adaptation, which aims to reduce a hunger stimulus by lowering plasma ghrelin concentrations under an energy surplus^31^, and the same phenomenon has been observed in people with binge eating^86,87^. The complicated ghrelin-GHSR system involves diverse hormonal signals, including gastrointestinal hormones, pancreatic hormones and multiple endocrine hormones^88^. Among the compensatory adaptations, the impact of glucose metabolism on energy homeostasis is well established. As a signal of positive energy, the increase in blood glucose stimulates the secretion of insulin and further suppresses ghrelin secretion, thus reducing plasma ghrelin levels^89,90^. In addition, recent studies have indicated that a positive energy balance impairs ghrelin’s functions in homeostatic feeding and reward processing, leading to a condition called ghrelin resistance, which reduces ghrelin action in the brain^26,91^. Based on the attenuated metabolic sensitivity, it is not surprising that the intervention of additional reduction or suppression of ghrelin provides limited efficacy. Moreover, the disruption of energy homeostasis in the higher body weight set-point may result in a compensatory increase in newly synthesized ghrelin, to say nothing of side effects relevant to glycemic control, accounting for prospects in animal experiments upon short-term use of ghrelin or GHSR antagonism, while long-term clinical efficacy has been minimal^49^.

Given the methodological differences in assay techniques or storage procedures, which are critical for the extremely susceptible ester bond of AG in the circulation^34^, subgroup analyses were conducted and showed robust decreased basal AG and DAG levels in obese patients compared with lean subjects for each stratification stratified by either the assay technique or storage procedure (Tables 2 and 3). According to the commercial recommendations, Acidification, low temperature, and enzymatic inhibitors were indispensable. Although we failed to detect the difference under different sample processing, the subgroup analyses illustrated the stable reduction of both AG and DAG in obesity.

### Postprandial acyl ghrelin in obesity

When comparing the different concentrations of postprandial AG between obese subjects and controls, the former still maintained significantly lower levels at each time stratification (Fig. 4 and Table 4). These results also reveal that a high level of ghrelin is not an inherent feature of simple obesity. We observed a postprandial decline in AG, both in healthy and obese individuals (Figs. 5, 6 and Table 5), although several studies demonstrated a temporary elevation after the initiation of an eating episode^72,92,93^. This inconsistency can be attributed to the different time points we selected because the rapid postprandial fall in circulating ghrelin levels is most likely to be triggered after nutrient ingestion^29^, even though macronutrient composition is taken into consideration^44^ and a postprandial response of plasma ghrelin requires postgastric stimulation. A longer gastric transition time might cause a longer duration for ghrelin suppression.

**Figure 8:**
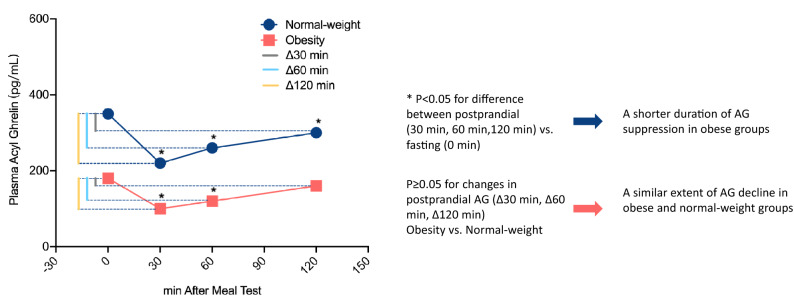
Diagram of the analysis of postprandial AG levels (the significance and AG levels reflect tendency only, cannot represent actual levels).

When stratified by the included time points, the difference in AG concentrations between postprandial 120 min and baseline states in obesity disappeared (SMD obtained from fixed-effects model: − 0.28; 95% CI: − 0.60 to 0.03; *P*_SMD_ = 0.074, Table 5), suggesting a shorter duration of AG suppression in obese subjects after meal intake because the difference was still significant in healthy controls at this time point (SMD obtained from fixed-effects model: − 0.42; 95% CI: − 0.77 to − 0.06; *P*_SMD_ = 0.021, Table 5). However, independent estimation of the extent of AG decline reached a similar value between the obese and healthy groups, since the mean change between baseline and postprandial states was not significantly different between the two groups in each period (Fig. 7 and Table 6), which means that obese subjects possess a similar degree of postprandial ghrelin reduction as normal weight subjects (Fig. 8).

To date, the study of the ghrelin response to meal in the obese subjects showed controversial results. Even existing researches prefer a blunted postprandial ghrelin suppression^33,59,94,95^, our finding is consistent with studies which do show a similarly meal-induced suppression between obesity and normal^44,96,97^. This phenomenon illustrates the establishment of a new body weight set-point and an adaptation of energy homeostasis under obese states. The shorter duration of AG suppression may be attributed to the lowering of basal ghrelin levels, rapidly recovering the starvation level, shortening the food-free interval between meals and causing frequent eating. In view of this faster rebound in postprandial suppression, it is not hard to understand the reversal of obesity-induced ghrelin suppression under calorie restriction^98^, and anti-ghrelin therapy may be more suitable for those recovery stages than for those lower baseline periods. More work is needed to fully elucidate ghrelin’s homeostasis, which will provide clues in therapeutic interventions for patients with metabolic diseases.

### Limitations

When applying the results in this meta-analysis, several limitations should be carefully considered. First, a relatively limited number of subjects were included in the evaluation of different forms of ghrelin independently between obese and lean healthy individuals, which might affect the statistical power. To expand the coverage of eligible studies, MetS patients were not excluded because abdominal obesity is one of the criteria to define metabolic syndrome^50,51^. However, metabolic comorbid conditions, including hypertension and IGT, could also affect ghrelin responses^99,100^. Second, the lack of sufficient data in these studies limited our further analysis, such as the postprandial DAG levels, AG/DAG ratio (a useful biomarker of excessive weight gain linked to obesity and diabetes), and AUC (area under the curve, an outcome representing overall hormone concentration over a specific time period in endocrinological studies). Furthermore, although Galbraith plots and subgroup analyses were used to explore heterogeneity, much of it remains to be explained and reported, including the varied types of mixed meals, different amounts of energy for meal tests, inconsistent duration of postprandial period, gender, ethnicity, age distribution and so on, and overestimating of pooled SMDs cannot be ignored. In addition, the language of the included studies was constrained to English, which was partially responsible for the publication biases.

## Conclusion

Taken together, our meta-analysis strengthens the clinical evidence supporting the following: lower baseline levels of circulating AG and DAG in obese individuals; the decline of postprandial circulating AG levels, both for healthy and obese individuals; and the shorter duration of AG suppression in obese subjects after meal intake. We support the existence of physiological adaptation in ghrelin under obesity, and the simultaneous decline in both AG and DAG is a symbol of positive energy balance. Despite some limitations in our study, we believe that this meta-analysis has significance for follow-up studies to elucidate the roles of various ghrelin forms in energy homeostasis. Furthermore, larger and more rigorous clinical trials with standardized test meals and fixed durations of the postprandial period are required to confirm these conclusions.

## Supplementary Information

Supplementary Information.

## Data Availability

No new data were created or analyzed in this study. Data sharing is not applicable to this article.
