# Supplementary material for: Circulating acyl and des-acyl ghrelin levels in obese adults: a systematic review and meta-analysis
Source: Sci Rep. 2022 Feb 17;12:2679. doi: 10.1038/s41598-022-06636-3 (PMC8854418; doi:10.1038/s41598-022-06636-3)
Supplement: Supplementary file 1 — Supplementary Information. [file 41598_2022_6636_MOESM1_ESM.docx]

Circulating acyl and des-acyl ghrelin levels in obese adults: A systematic review and meta-analysis

Yanmei Wang^1,2#^, Qianxian Wu^2#^, Qian Zhou^1^, Yuyu Chen^3^, Xingxing Lei^1^, Yiding Chen^1^, Qiu Chen^1*^

^1^Hospital of Chengdu University of Traditional Chinese Medicine, No. 39 Shi-er-qiao Road, Jinniu District, Chengdu 610075, Sichuan, China
^2^Ya‘an Polytechnic college, No. 130 Yucai Road, Yucheng District, Yaan 625000, Sichuan, China
^3^Halifa Regional Centre for Education, No. 33 Spectacle Lake Dr, Dartmouth, B3B1X7, Nova Scotia, Canada
^*^corresponding. [chenqiu1005@cdutcm.edu.cn](mailto:chenqiu1005@cdutcm.edu.cn)

^#^contributed equally to this work.

Supplementary Materials

Supplementary Table 1. Search strategy

| Set | Search terms (read from bottom-up) |
| --- | --- |
| PubMed October 22th, 2021 | |
| #3 | #1 AND #2 |
| #2 | desacylghrelin OR 'desacyl ghrelin' OR 'des-acyl ghrelin' OR 'des-n-octanoyl ghrelin' OR 'unacyl ghrelin' OR 'unacyl-ghrelin' OR 'acyl ghrelin' OR 'acyl-ghrelin' OR acylghrelin OR 'active ghrelin' OR ‘active-ghrelin’ OR ‘acylated ghrelin’ OR ‘acylated-ghrelin’ OR ‘unacylated ghrelin’ OR ’unacylated-ghrelin’ OR ’des-acylated ghrelin’ OR ‘desacylated-ghrelin’ OR ‘desacylated ghrelin’ OR ’non-aclyated ghrelin’ OR ‘nonacylated ghrelin’ OR ‘nonacylated-ghrelin’ |
| #1 | obesity[Mesh] OR 'weight gain' OR overweight[Mesh] OR adiposity OR 'body fat distribution' OR 'waist circumference' OR 'body mass index' OR 'intra-abdominal fat' OR 'abdominal fat' OR obese OR 'abdominal circumference' OR 'body weight' OR overnutrition OR 'anthropometric measurement' OR 'anthropometric measurements' OR 'anthropometric measure' OR 'adipose tissue hyperplasia' OR 'adipositas' OR 'alimentary obesity' OR 'corpulency' OR 'fat overload syndrome' OR 'nutritional obesity' OR obesitas |
| Embase October 22th, 2021e | |
| #3 | #1 AND #2 |
| #2 | desacylghrelin OR 'desacyl ghrelin' OR 'des-acyl ghrelin' OR 'des-n-octanoyl ghrelin' OR 'unacyl ghrelin' OR 'unacyl-ghrelin' OR 'acyl ghrelin' OR 'acyl-ghrelin' OR acylghrelin OR 'active ghrelin' OR ‘active-ghrelin’ OR ‘acylated ghrelin’ OR ‘acylated-ghrelin’ OR ‘unacylated ghrelin’ OR ‘unacylated-ghrelin’ OR ‘des-acylated ghrelin’ OR ‘desacylated-ghrelin’ OR ‘desacylated ghrelin’ OR ‘non-aclyated ghrelin’ OR ‘nonacylated ghrelin’ OR ‘nonacylated-ghrelin’ |
| #1 | 'obesity'/exp OR 'weight gain' OR overweight OR adiposity OR 'body fat distribution' OR 'waist circumference' OR 'body mass index' OR 'intra-abdominal fat' OR 'abdominal fat' OR obese OR 'abdominal circumference' OR 'body weight' OR overnutrition OR 'anthropometric measurement' OR 'anthropometric measurements' OR 'anthropometric measure' OR 'adipose tissue hyperplasia' OR 'adipositas' OR 'alimentary obesity' OR corpulency OR 'fat overload syndrome' OR 'nutritional obesity' OR obesitas |
| Web Of Science October 22th, 2021 | |
| #3 | #1 AND #2 |
| #2 | TS=(desacylghrelin OR 'desacyl ghrelin' OR 'des-acyl ghrelin' OR 'des-n-octanoyl ghrelin' OR 'unacyl ghrelin' OR 'unacyl-ghrelin' OR 'acyl ghrelin' OR 'acyl-ghrelin' OR acylghrelin OR  'active ghrelin' OR 'active-ghrelin' OR ‘acylated ghrelin’ OR ‘acylated-ghrelin’ OR ‘unacylated ghrelin’ OR ‘unacylated-ghrelin’ OR ‘des-acylated ghrelin’ OR ‘desacylated-ghrelin’ OR ‘desacylated ghrelin’ OR ‘non-aclyated ghrelin’ OR ‘nonacylated ghrelin’ OR ‘nonacylated-ghrelin’) |
| #1 | TS=(“weight gain” OR obesity OR overweight OR adiposity OR “body fat distribution” OR “waist circumference” OR “body mass index” OR “intra-abdominal fat” OR “abdominal fat” OR obese OR “abdominal circumference” OR “body weight” OR overnutrition OR “anthropometric measurement” OR “anthropometric measurements” OR “anthropometric measure”OR 'adipose tissue hyperplasia' OR 'adipositas' OR 'alimentary obesity' OR 'corpulency' OR 'fat overload syndrome' OR 'nutritional obesity' OR 'obesitas' ) |

Supplementary Table 2 Summary of circulating ghrelin concentrations of the included studies

| Reference | Group | Fasting | | Postprandial 30 min | Postprandial 60 min | | Postprandial 120 min | unit |
| --- | --- | --- | --- | --- | --- | --- | --- | --- |
|  |  | AG | DAG | AG | AG | | AG |  |
| B. Baranowska 2006^1^ | Control | 85.6± 62.1 | NR | NR | NR | | NR | pmol/L |
|  | Obese | 49.8±55.3 | NR | NR | NR | | NR |  |
| H. M. Homaee 2011^2^ | Control | 241.8±21.4 | NR | NR  NR | NR  NR | | NR  NR | pg/mL |
|  | Obese | 110.5±18.5 | NR |  |  |  |  |  |
| S. Iceta 2019^3^ | Control | 107.0±64.6 | 365.0±172.3 | NR  NR | NR  NR | | NR  NR | pg/mL |
|  | Obese | 36.0±44.5 | 145.0±111.2 |  |  |  |  |  |
| P. A. Kołodziejski 2018^4^ | Control | 24.7±13.4 | 1324.3±481.5 | NR  NR | NR  NR | | NR  NR | pg/mL |
|  | Obese | 36.9±10.18 | 1637.1±408.2 |  |  |  |  |  |
| T. Nakahara 2008^5^ | Control | 27.0±10.3 | 261.7±119.1 | NR  NR | NR  NR | | NR  NR | fmol/L |
|  | Obese | 13.0±7.3 | 145.1±41.4 |  |  |  |  |  |
| S. Ezquerro 2019^6^ | Control | 8.3±4.4 | 68.2±51.5 | NR  NR  NR | NR  NR  NR | | NR  NR  NR | pmol/L |
|  | Obese-NG | 5.1±5.4 | 41.8±5.3 |  |  |  |  |  |
|  | Obese-IGT | 4.9±3.9 | 33.6±4.5 |  |  |  |  |  |
| D. Haluzíková 2013^7^ | Control | 113.5±40.0 | NR | NR  NR | NR  NR | | NR  NR | pg/mL |
|  | Obese | 55.8±58.9 | NR |  |  |  |  |  |
| R. A. Tamboli 2017^8^ | Control | 79.8±57.0 | 674.2±208.4 | NR  NR | NR  NR | | NR  NR | pg/mL |
|  | Obese | 31.7±10.8 | 316.3±115.0 |  |  |  |  |  |
| S. W. Savage 2014^9^ | Control | 245.1±90.7 | NR | NR  NR | NR  NR | | NR  NR | pg/mL |
|  | Obese | 121.9±207.1 | NR |  |  |  |  |  |
| A. M. Arafat 2013^10^ | Control | 346.0±238.0 | 866.2±485.1 | NR | NR | | NR | pg/mL |
|  | Obese | 220.7±122.4 | 502.3±158.5 | NR | | NR | NR |  |
| A. Rodríguez 2012^11^ | Control | 3.9±2.2 | 168.1±115.7 | NR | | NR | NR | pmol/L |
|  | Obese-NG | 4.6±2.7 | 90.7±57.7 | NR | | NR | NR |  |
|  | Obese-IGT | 4.4±2.6 | 97.7±57.4 | NR | | NR | NR |  |

| Supplementary Table 2(Continued) | |  |  |  |  |  |  |
| --- | --- | --- | --- | --- | --- | --- | --- |
| Reference | Group | Fasting | | Postprandial 30 min | Postprandial 60 min | Postprandial 120 min | unit |
|  |  | AG | DAG | AG | AG | AG |  |
| J. P. Dunn 2012^12^ | Control | 235.0±104.0 | NR | NR | NR | NR | pg/mL |
|  | Obese | 105.0±57.0 | NR | NR | NR | NR |  |
| J. F. Carroll 2009^13^ | Control | 89.3±47.6 | NR | NR | NR | NR | pg/mL |
|  | Obese | 46.5±22.5 | NR | NR | NR | NR |  |
| P. Marzullo 2004^14^ | Control | 411.8±256.7 | 5256.2±2760.7 | NR | NR | NR | pg/mL |
|  | Obese | 180.4±82.7 | 3470.6±1784.7 | NR | NR | NR |  |
| M. Suematsu 2005^15^ | Control | 40.9±16.1 | NR | NR | NR | NR | fmol/L |
|  | Obese | 20.4±10.7 | NR | NR | NR | NR |  |
| W. Bik 2007^16^ | Control | 85.2±67.1 | NR | NR | NR | NR | pg/mL |
|  | Obese | 49.7±57.2 | NR | NR | NR | NR |  |
| P. Marzullo 2008^17^ | Control | 410.0±59.4 | 4126.0±238.9 | NR | NR | NR | pg/mL |
|  | Obese | 290.0±121.6 | 2763.0±836.8 | NR | NR | NR |  |
| A. G. Yunker 2021^18^ | Control | 154.7±86.9 | NR | NR | NR | NR | pg/mL |
|  | Over weight | 117.0±63.2 | NR | NR | NR | NR |  |
|  | Obese | 95.5±56.9 | NR | NR | NR | NR |  |
| J. P. Nogueira 2012^19^ | Control | NR | 298.6±99.4 | NR | NR | NR | pg/mL |
|  | Obese-low HDL-c | NR | 161.2±49.0 | NR | NR | NR |  |
|  | Obese-MetS | NR | 150.3±61.6 | NR | NR | NR |  |
|  | Obese | NR | 185.3±85.3 | NR | NR | NR |  |
| I. Lopez-Aguilar 2018^20^ | Control | 52.9±42.2 | NR | NR | NR | NR | pg/mL |
|  | Obese | 35.3±33.3 | NR | NR | NR | NR |  |
| Y. Ozkan 2013^21^ | Low weight | 16.0±11.1 | NR | NR | NR | NR | pg/mL |
|  | Normal weight | 16.1±5.8 | NR | NR | NR | NR |  |
|  | Over weight | 16.8±4.1 | NR | NR | NR | NR |  |
|  | Obese | 19.6±3.6 | NR | NR | NR | NR |  |
|  | Morbidly obese | 19.7±5.6 | NR | NR | NR | NR |  |

| Supplementary Table 2(Continued) | |  |  |  |  |  |  |
| --- | --- | --- | --- | --- | --- | --- | --- |
| Reference | Group | Fasting | | Postprandial 30 min | Postprandial 60 min | Postprandial 120 min | unit |
|  |  | AG | DAG | AG | AG | AG |  |
| R. Gelisgen 2012^22^ | Control | 5.3±4.8 | NR | NR | NR | NR | ng/mL |
|  | Morbidly obese | 2.8±4.5 | NR | NR | NR | NR |  |
| I. Karcz-Socha 2011^23^ | Control | 165.4±53.6 | 624.6±140.4 | NR | NR | NR | pg/mL |
|  | Moderately obese | 161.7±58.3 | 383.1±137.9 | NR | NR | NR |  |
|  | Morbidly obese | 143.0±60.7 | 343.8±133.5 | NR | NR | NR |  |
| B. Krzyzanowska-Swiniarska 2007^24^ | Control | 22.1±8.5 | NR | NR | NR | NR | pg/mL |
|  | Obese without  insulin resistance | 21.7±11.6 | NR | NR | NR | NR |  |
|  | Obese with insulin resistance | 21.9±7.5 | NR | NR | NR | NR |  |
| K. Zwirska-Korczala 2007^25^ | Control | 199.0±65.1 | 651.0±218.1 | NR | NR | NR | pg/mL |
|  | Moderately obese-MetS | 108.0±41.6 | 447.0±214.4 | NR | NR | NR |  |
|  | Morbidly obese-MetS | 194.0±111.3 | 507.0±282.9 | NR | NR | NR |  |
| E. P. Rizi,2018^26^ | Control | 328.3±208.4 | NR | 307.1±251.6 | 235.9±159.8 | 217.5±146.0 | pg/mL |
|  | Obese | 181.3±112.9 | NR | 143.9±74.4 | 133.6±65.1 | 119.7±66.6 |  |
| S. Brede 2017^27^ | Control | 69.6±25.9 | 69.6±25.9 | 40.3±12.6 | NR | NR | pg/mL |
|  | Obese | 38.3±13.1 | 38.3±13.1 | 29.2±10.4 | NR | NR |  |
| J. A. Douglas 2017^28^ | Control | 104.0±58.0 | 241.0±131.0 | 58.4±35.1 | 48.4±58.9 | NR | pg/mL |
|  | Obese | 122.0±100.0 | 349.0±381.0 | 62.2±92.7 | 53.8±54.1 | NR |  |
| K. Seyssel 2016^29^ | Control | 74.5±45.2 | NR | 58.0±49.8 | NR | NR | pg/mL |
|  | Obese | 29.1±18.1 | NR | 16.4±15.0 | NR | NR |  |
| J. A. Dardzińska 2014^30^ | Control | 148.0±56.0 | 583.0±369.0 | NR | NR | 122.0±66.0 | pg/mL |
|  | Obese | 44.0±26.0 | 287.0±219.0 | NR | NR | 41.0±23.0 |  |

| Supplementary Table 2(Continued) | |  |  |  |  |  |  |
| --- | --- | --- | --- | --- | --- | --- | --- |
| Reference | Group | Fasting | | Postprandial 30 min | Postprandial 60 min | Postprandial 120 min |  |
|  |  | AG | DAG | AG | AG | AG |  |
| T. D. Heden 2013^31^ | Control | 114.0±63.6 | NR | 66.4±43.4 | 56.5±54.6 | 77.0±54.6 | pg/mL |
|  | Obese | 75.0±63.6 | NR | 44.4±36.9 | 35.8±29.6 | 47.9±40.9 |  |
| S. Y. Ueda 2009^32^ | Control | 108.2±53.1 | NR | NR | 28.6±14.8 | 48.2±25.8 | pmol/L |
|  | Obese | 127.9±37.8 | NR | NR | 62.4±41.6 | 67.1±42.8 |  |
| N. Tentolouris 2004^33^ | Control | 224.9±123.3 | NR | NR | 189.1±102.2 | 217.1±116.9 | pmol/L |
|  | Obese | 68.4±34.6 | NR | NR | 65.6±45.8 | 71.1±51.3 |  |
| D. Foschi 2005^34^ | Control | 130.1±5.9 | NR | NR | 117.4±14.0 | 138.0±30.9 | pg/mL |
|  | Obese | 97.2±30.0 | NR | NR | 91.7±28.8 | 96.7±49.0 |  |
| Data are presented as the means ± SD; MetS: metabolic syndrome; HDL-c: High-density lipoprotein-cholesterol; NG: normoglycemia; IGT: impaired glucose tolerance; AG: acyl ghrelin; DAG: des-acyl ghrelin; NR: not reported | | | | | | | |

#

# Supplementary Figure 1. Quality assessment of included articles according to The

# Newcastle-Ottawa Quality Assessment Scale. +: YES; -: NO; ? : Cannot say.

**
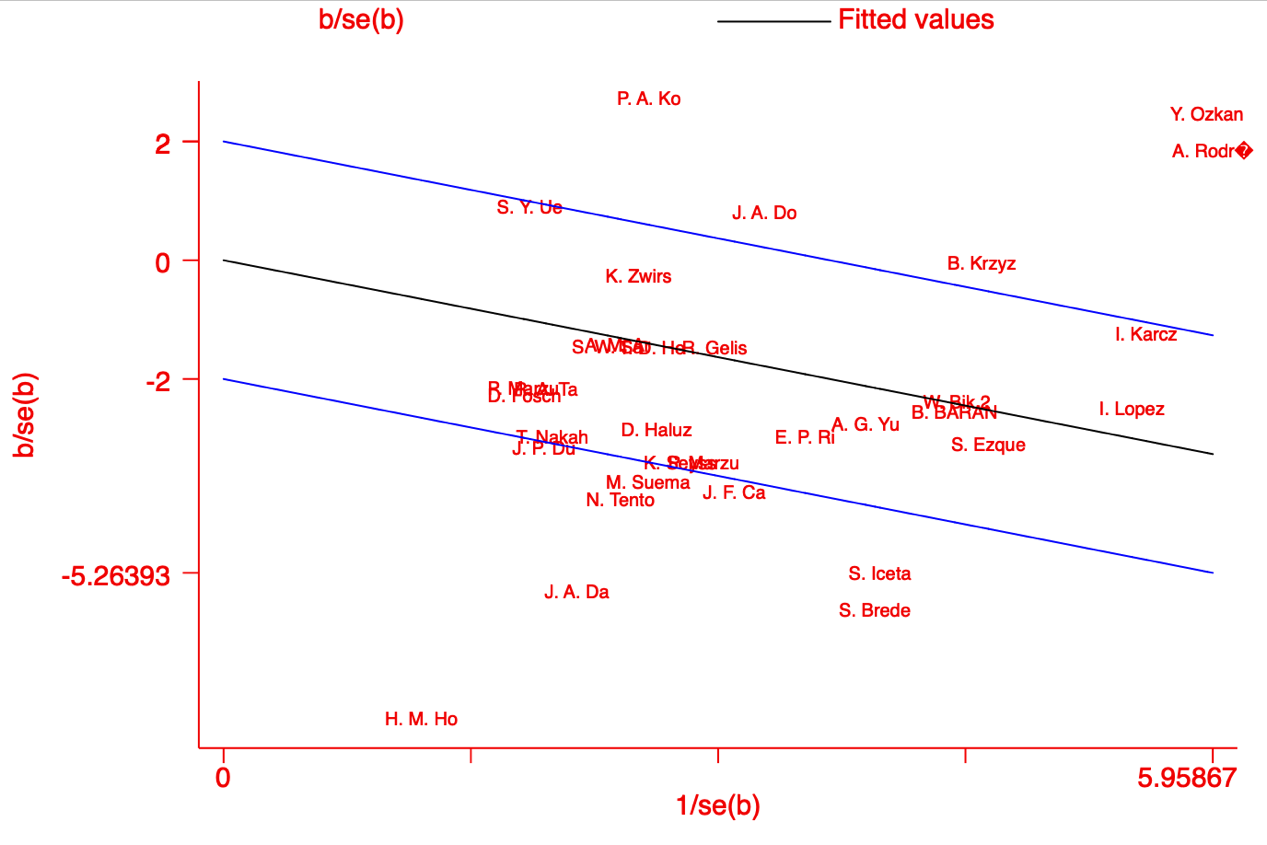
**

# Supplementary Figure 2. Galbraith plot for comparisons of fasting AG levels

**
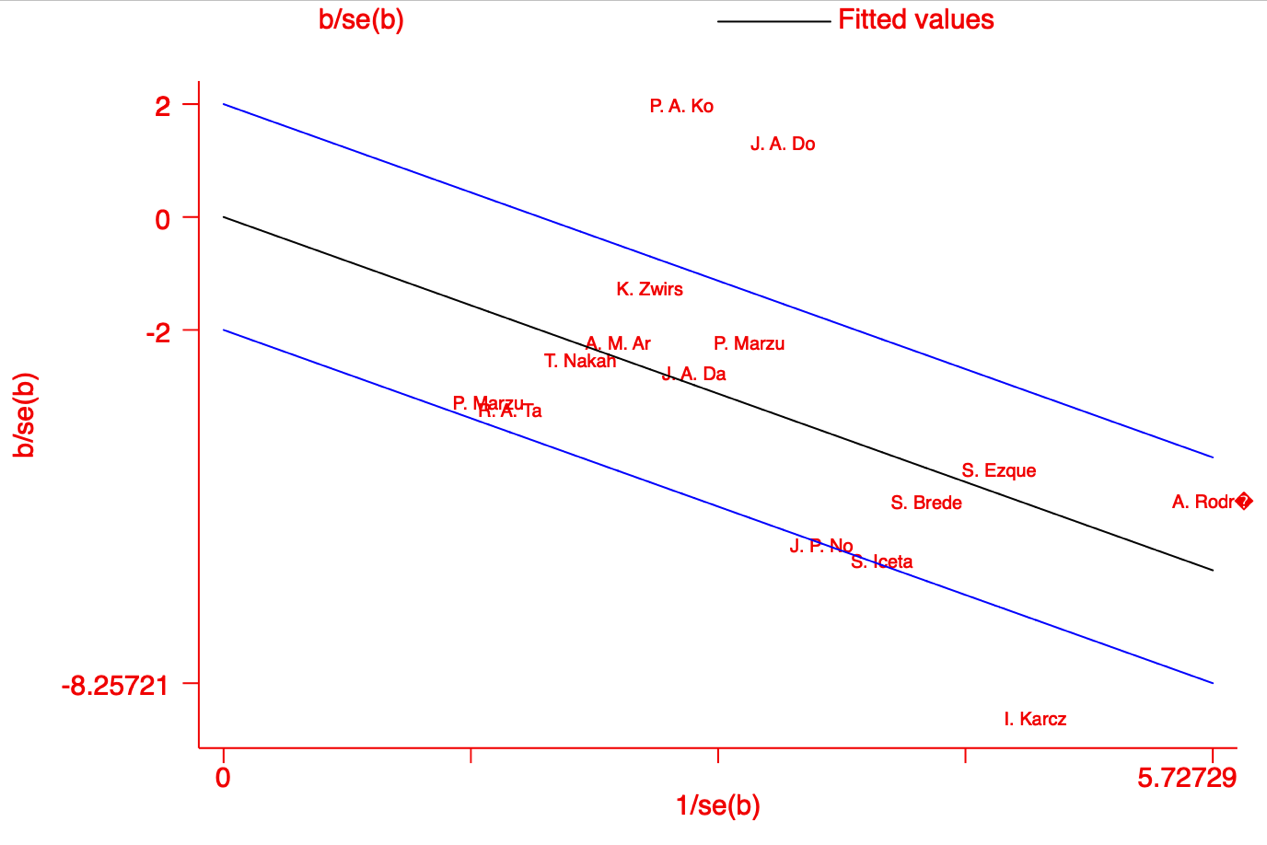
**

# Supplementary Figure 3. Galbraith plot for comparisons of fasting DAG levels


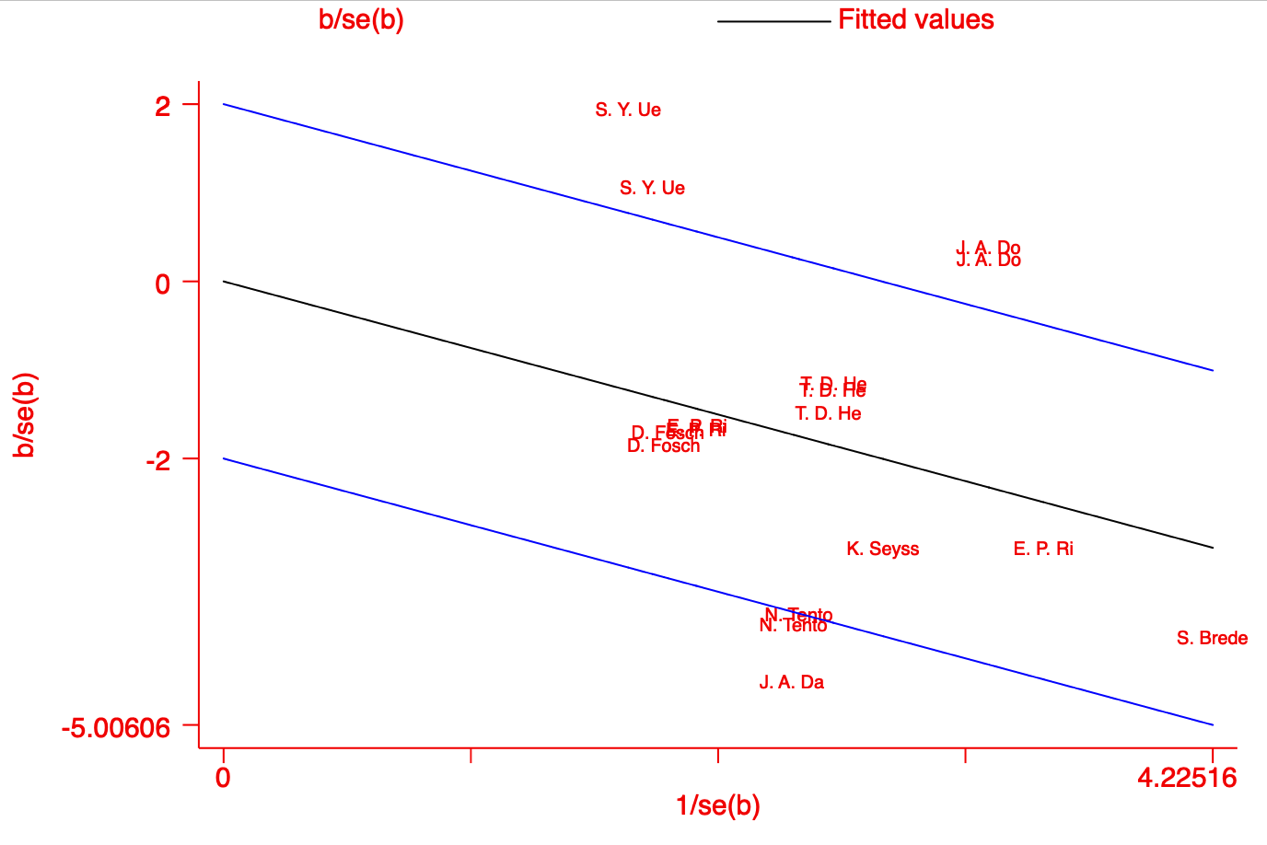


# Supplementary Figure 4. Galbraith plot for comparisons of postprandial AG levels (obesity vs. normal-weight)


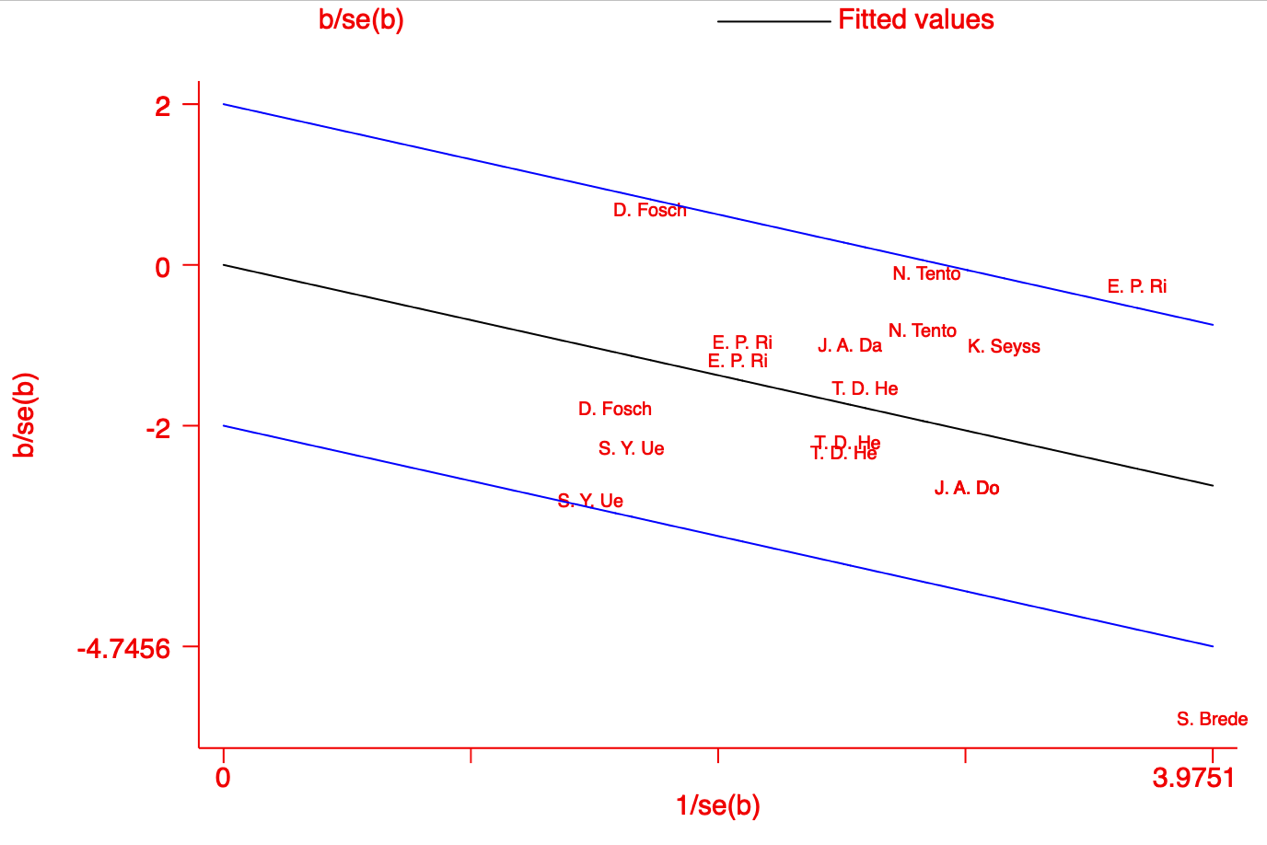


Supplementary Figure 5. Galbraith plot for comparisons of postprandial AG levels in normal-weight group (postprandial vs. baseline)


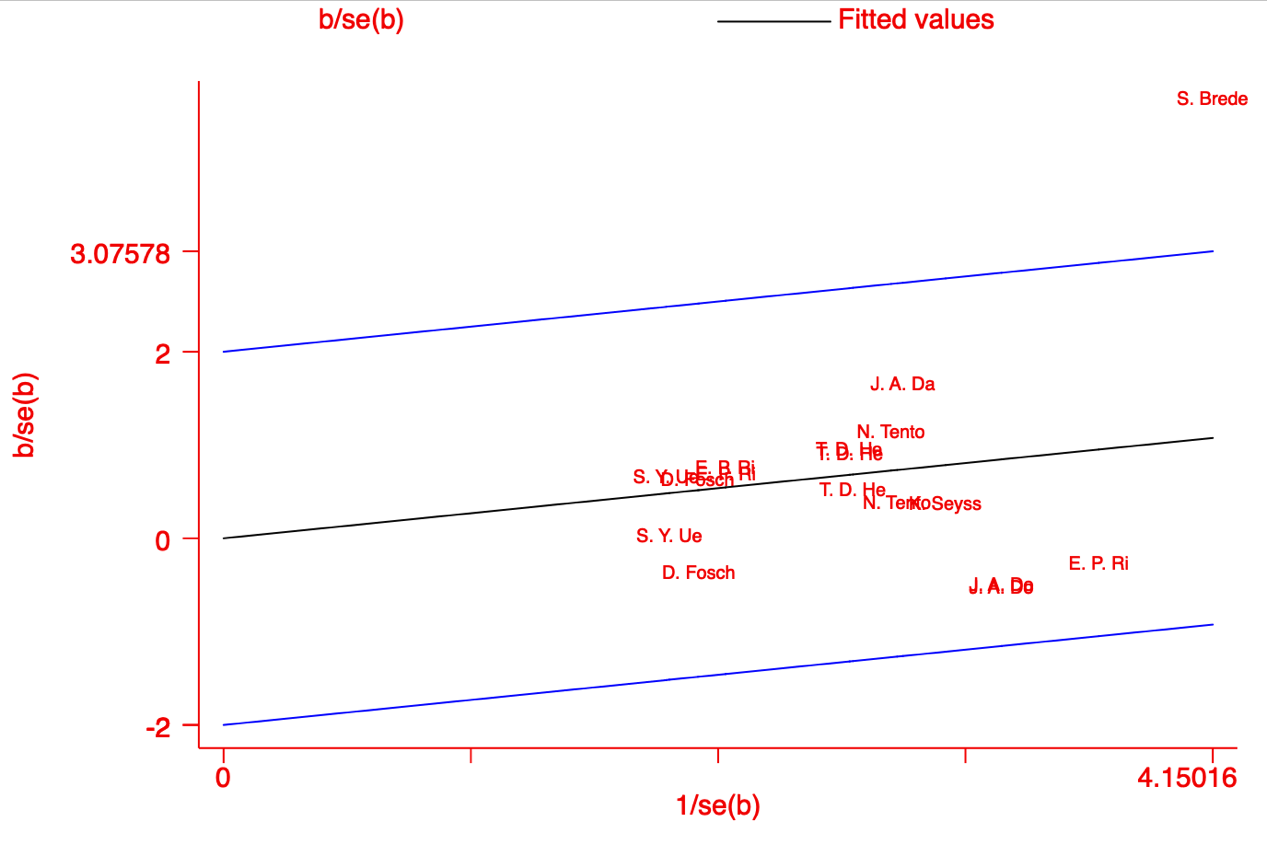


Supplementary Figure 6. Galbraith plot of the changes in postprandial AG levels


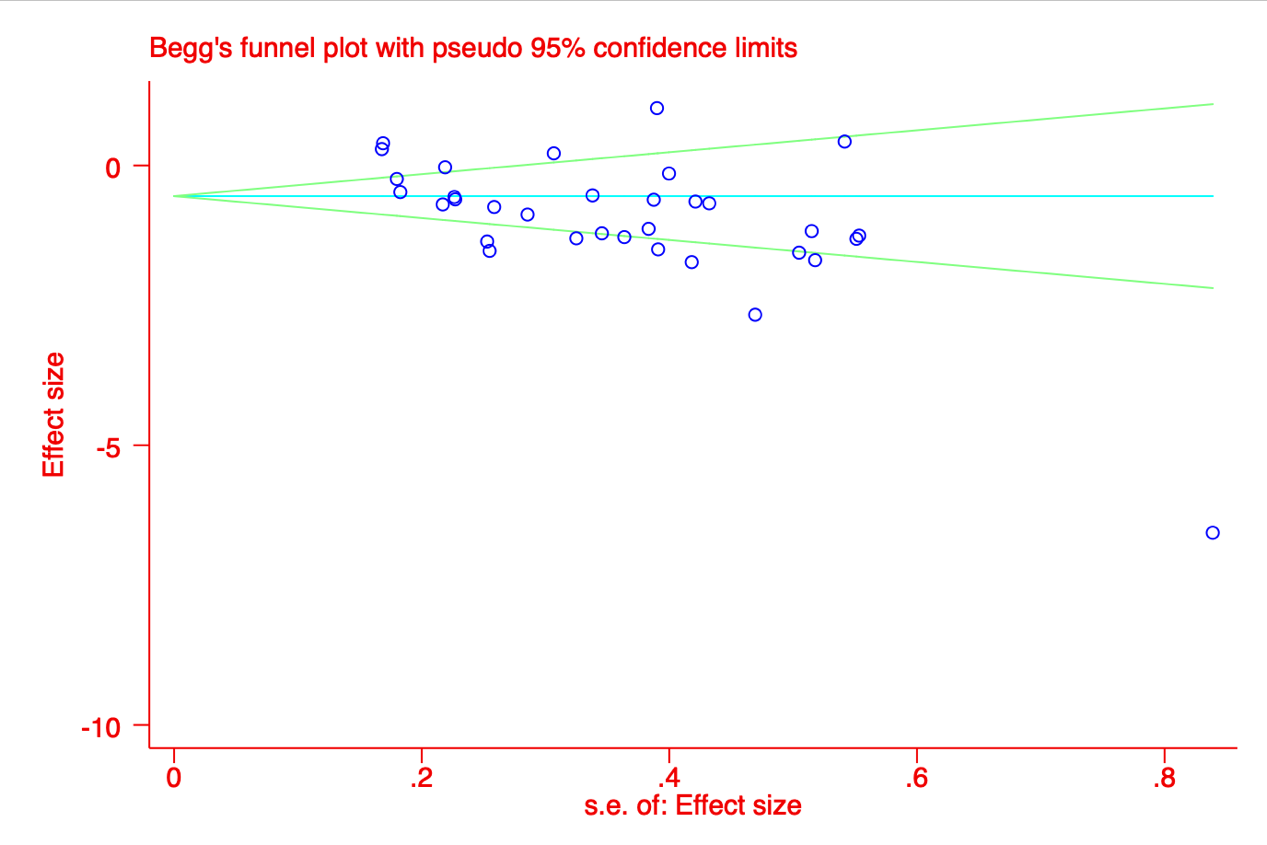


# Supplementary Figure 7. Begg’s funnel plot for comparisons of fasting AG levels

#
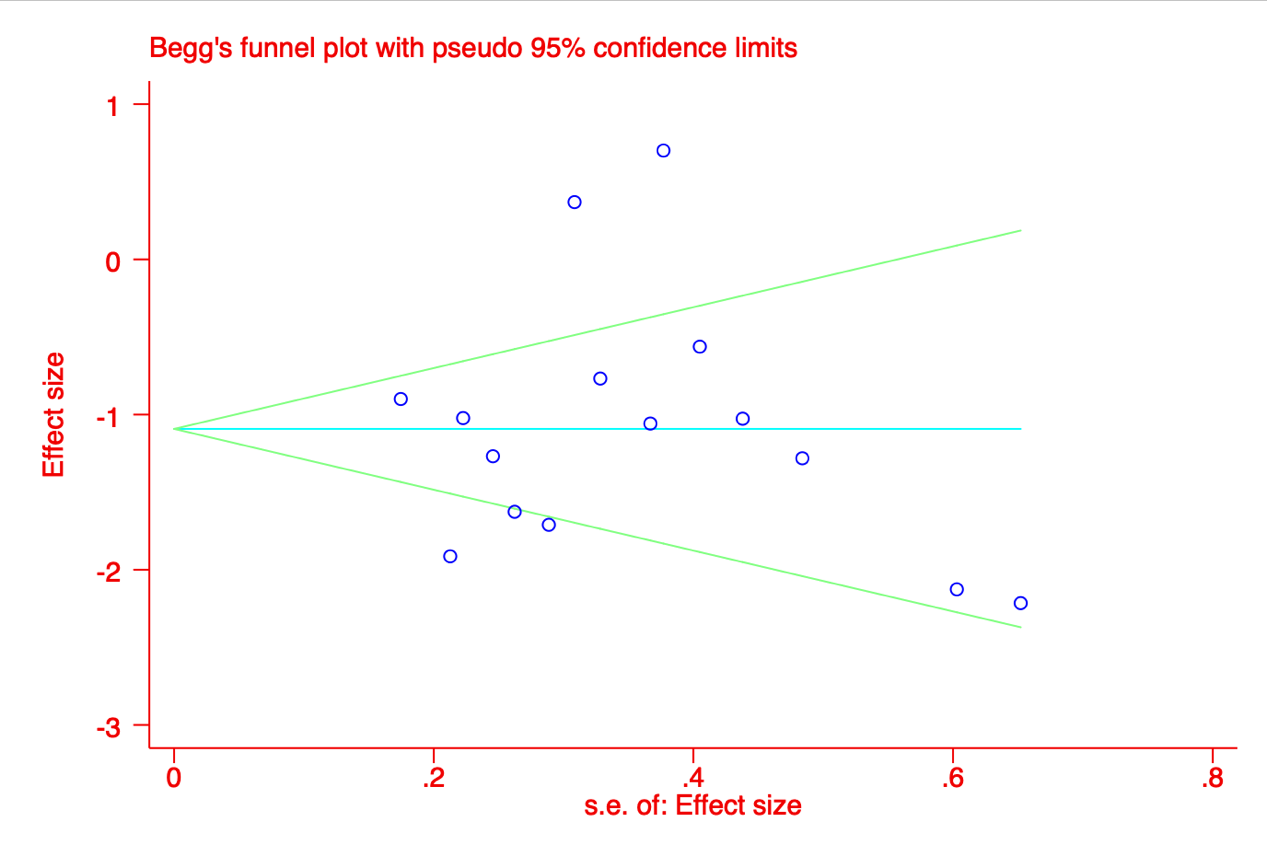


# Supplementary Figure 8. Begg’s funnel plot for comparisons of fasting DAG levels

### References

1 Baranowska, B. *et al.* Neuroendocrine control of metabolic homeostasis in Polish centenarians. *J Physiol Pharmacol* **57**, 55-61, doi:10.1152/jn.00538.2006 (2006).

2 Homaee, H. M., Moradi, F., Azarbayjani, M. A. & Peeri, M. Relationships between acylated ghrelin with growth hormone, insulin resistance, lipid profile, and cardio respiratory function in lean and obese men. *J Res Med Sci* **16**, 1612-1618 (2011).

3 Iceta, S. *et al.* Ghrelin concentration as an indicator of eating-disorder risk in obese women. *Diabetes and Metabolism* **45**, 160-166, doi:10.1016/j.diabet.2018.01.006 (2019).

4 Kołodziejski, P. A. *et al.* Serum levels of spexin and kisspeptin negatively correlate with obesity and insulin resistance in women. *Physiological research* **67**, 45-56, doi:10.33549/physiolres.933467 (2018).

5 Nakahara, T. *et al.* Plasma Obestatin Concentrations Are Negatively Correlated with Body Mass Index, Insulin Resistance Index, and Plasma Leptin Concentrations in Obesity and Anorexia Nervosa. *Biological Psychiatry* **64**, 252-255, doi:10.1016/j.biopsych.2007.08.005 (2008).

6 Ezquerro, S. *et al.* Ghrelin Reduces TNF-alpha-Induced Human Hepatocyte Apoptosis, Autophagy, and Pyroptosis: Role in Obesity-Associated NAFLD. *Journal of Clinical Endocrinology & Metabolism* **104**, 21-37, doi:10.1210/jc.2018-01171 (2019).

7 Haluzíková, D. *et al.* Laparoscopic sleeve gastrectomy differentially affects serum concentrations of FGF-19 and FGF-21 in morbidly obese subjects. *Obesity (Silver Spring)* **21**, 1335-1342, doi:10.1002/oby.20208 (2013).

8 Tamboli, R. A. *et al.* Metabolic responses to exogenous ghrelin in obesity and early after Roux-en-Y gastric bypass in humans. *Diabetes, Obesity and Metabolism* **19**, 1267-1275, doi:10.1111/dom.12952 (2017).

9 Savage, S. W. *et al.* Regulation of novelty seeking by midbrain dopamine D2/D3 signaling and ghrelin is altered in obesity. *Obesity* **22**, 1452-1457, doi:10.1002/oby.20690 (2014).

10 Arafat, A. M. *et al.* The impact of insulin-independent, glucagon-induced suppression of total ghrelin on satiety in obesity and type 1 diabetes mellitus. *Journal of Clinical Endocrinology and Metabolism* **98**, 4133-4142, doi:10.1210/jc.2013-1635 (2013).

11 Rodríguez, A. *et al.* The ghrelin O-Acyltransferase-Ghrelin system reduces TNF-α-Induced apoptosis and autophagy in human visceral adipocytes. *Diabetologia* **55**, 3038-3050, doi:10.1007/s00125-012-2671-5 (2012).

12 Dunn, J. P. *et al.* Relationship of dopamine type 2 receptor binding potential with fasting neuroendocrine hormones and insulin sensitivity in human obesity. *Diabetes Care* **35**, 1105-1111, doi:10.2337/dc11-2250 (2012).

13 Carroll, J. F., Franks, S. F., Smith, A. B. & Phelps, D. R. Visceral adipose tissue loss and insulin resistance 6 months after laparoscopic gastric banding surgery: a preliminary study. *Obes Surg* **19**, 47-55, doi:10.1007/s11695-008-9642-4 (2009).

14 Marzullo, P. *et al.* The Relationship between Active Ghrelin Levels and Human Obesity Involves Alterations in Resting Energy Expenditure. *Journal of Clinical Endocrinology and Metabolism* **89**, 936-939, doi:10.1210/jc.2003-031328 (2004).

15 Suematsu, M. *et al.* Decreased circulating levels of active ghrelin are associated with increased oxidative stress in obese subjects. *European Journal of Endocrinology* **153**, 403-407, doi:10.1530/eje.1.01977 (2005).

16 Bik, W. *et al.* The relationship between metabolic status and levels of adiponectin and ghrelin in lean women with polycystic ovary syndrome. *Gynecological Endocrinology* **23**, 325-331, doi:10.1080/09513590701260169 (2007).

17 Marzullo, P. *et al.* Acylated ghrelin decreases during acute exercise in the lean and obese state. *Clin Endocrinol (Oxf)* **69**, 970-971, doi:10.1111/j.1365-2265.2008.03275.x (2008).

18 Yunker, A. G. *et al.* Appetite-Regulating Hormones Are Reduced After Oral Sucrose vs Glucose: Influence of Obesity, Insulin Resistance, and Sex. *The Journal of clinical endocrinology and metabolism* **106**, 654-664, doi:10.1210/clinem/dgaa865 (2021).

19 Nogueira, J. P. *et al.* Unacylated Ghrelin is associated with the isolated low HDL-cholesterol obese phenotype independently of insulin resistance and CRP level. *Nutr Metab (Lond)* **9**, 17, doi:10.1186/1743-7075-9-17 (2012).

20 Lopez-Aguilar, I., del Rocio Ibarra-Reynoso, L. & Manuel Malacara, J. Association of Nesfatin-1, Acylated Ghrelin and Cortisol with Scores of Compulsion, Food Addiction, and Binge Eating in Adults with Normal Weight and with Obesity. *Annals of Nutrition and Metabolism* **73**, 54-61, doi:10.1159/000490357 (2018).

21 Ozkan, Y. *et al.* Acylated and desacylated ghrelin, preptin, leptin, and nesfatin-1 Peptide changes related to the body mass index. *Int J Endocrinol* **2013**, 236085, doi:10.1155/2013/236085 (2013).

22 Gelisgen, R. *et al.* Effects of Laparoscopic Gastric Band Applications on Plasma and Fundic Acylated Ghrelin Levels in Morbidly Obese Patients. *Obesity Surgery* **22**, 299-305, doi:10.1007/s11695-011-0498-7 (2012).

23 Karcz-Socha, I., Zwirska-Korczala, K., Zembala, M., Borgiel-Marek, H. & Karcz, W. K. Ghrelin PYY 3-36 Serum Changes in Left Ventricular Hypertrophic, Insulin-Resistant, Hypertensive Obese Patients. *Obesity Facts* **4**, 386-392, doi:10.1159/000334198 (2011).

24 Krzyzanowska-Swiniarska, B., Kempa, A., Miazgowski, T. & Pilarska, K. Serum acylated ghrelin, adiponectin and leptin levels in normal-weight and obese premenopausal women. *Horm Metab Res* **39**, 835-839, doi:10.1055/s-2007-991175 (2007).

25 Zwirska-Korczala, K. *et al.* Basal and postprandial plasma levels of PYY, ghrelin, cholecystokinin, gastrin and insulin in women with moderate and morbid obesity and metabolic syndrome. *Journal of Physiology and Pharmacology* **58**, 13-35 (2007).

26 Rizi, E. P. *et al.* A high carbohydrate, but not fat or protein meal attenuates postprandial ghrelin, PYY and GLP-1 responses in Chinese men. *PLoS ONE* **13**, 1-12, doi:10.1371/journal.pone.0191609 (2018).

27 Brede, S. *et al.* Visual food cues decrease postprandial glucose concentrations in lean and obese men without affecting food intake and related endocrine parameters. *Appetite* **117**, 255-262, doi:10.1016/j.appet.2017.07.001 (2017).

28 Douglas, J. A. *et al.* Acute effects of exercise on appetite, ad libitum energy intake and appetite-regulatory hormones in lean and overweight/obese men and women. *Int J Obes (Lond)* **41**, 1737-1744, doi:10.1038/ijo.2017.181 (2017).

29 Seyssel, K. *et al.* Plasma acyl-ghrelin increases after meal initiation: A new insight. *European Journal of Clinical Nutrition* **70**, 790-794, doi:10.1038/ejcn.2015.181 (2016).

30 Dardzińska, J. *et al.* Fasting and postprandial acyl and desacyl ghrelin levels in obese and non-obese subjects. *Endokrynologia Polska* **65**, 377-381, doi:10.5603/ep.2014.0052 (2014).

31 Heden, T. D., Liu, Y., Park, Y., Dellsperger, K. C. & Kanaley, J. A. Acute aerobic exercise differentially alters acylated ghrelin and perceived fullness in normal-weight and obese individuals. *J Appl Physiol (1985)* **115**, 680-687, doi:10.1152/japplphysiol.00515.2013 (2013).

32 Ueda, S. Y. *et al.* Changes in gut hormone levels and negative energy balance during aerobic exercise in obese young males. *J Endocrinol* **201**, 151-159, doi:10.1677/joe-08-0500 (2009).

33 Tentolouris, N. *et al.* Differential effects of high-fat and high-carbohydrate content isoenergetic meals on plasma active ghrelin concentrations in lean and obese women. *Hormone and Metabolic Research* **36**, 559-563, doi:10.1055/s-2004-825761 (2004).

34 Foschi, D. *et al.* Vertical banded gastroplasty modifies plasma ghrelin secretion in obese patients. *Obesity Surgery* **15**, 1129-1132, doi:10.1381/0960892055002338 (2005).
